# Interconnected marine habitats form a single continental-scale reef system in South America

**DOI:** 10.1038/s41598-022-21341-x

**Published:** 2022-10-17

**Authors:** Pedro B. M. Carneiro, Antônio R. Ximenes Neto, Bruno Jucá-Queiroz, Carlos E. P. Teixeira, Caroline V. Feitosa, Cristiane X. Barroso, Helena Matthews-Cascon, Jader O. de Morais, João E. P. Freitas, Jones Santander-Neto, Jorge T. de Araújo, Leonardo H. U. Monteiro, Lidriana S. Pinheiro, Marcus D. A. Braga, Ralf T. S. Cordeiro, Sergio Rossi, Sonia Bejarano, Sula Salani, Tatiane M. Garcia, Tito M. C. Lotufo, Tyler B. Smith, Vicente V. Faria, Marcelo O. Soares

**Affiliations:** 1 Universidade Federal do Delta do Parnaíba, Av. São Sebastião 2819, Campus Ministro Reis Velloso, Parnaíba, PI 64202-020 Brazil; 2 LGCO, Universidade Estadual do Ceará, Av. Dr. Silas Munguba 1700, Campus do Itaperi, Fortaleza, CE 60714-903 Brazil; 3 Departamento de Biologia, Universidade Federal do Ceará, Av. Mister Hull s/n, Campus do Pici, Fortaleza, CE 60440-900 Brazil; 4 Instituto de Ciências do Mar (LABOMAR), Universidade Federal do Ceará, Av. Abolição 3207, Fortaleza, CE 60165-081 Brazil; 5 Instituto Federal de Educação, Ciência e Tecnologia do Espírito Santo, R. Augusto Costa de Oliveira 660, Campus Piúma, Piúma, ES 29285-000 Brazil; 6 IVIG, COPPE, Universidade Federal Do Rio de Janeiro, Cidade Universitária, Rio de Janeiro, RJ 21941-914 Brazil; 7 Grupo Sandmine & Inframar, R. Pedro Rufino 90, Fortaleza, CE 60175-100 Brazil; 8 Mar do Ceará, LTDA., Av. João Pessoa 5834 sala 01, Fortaleza, CE 60420-680 Brazil; 9 Departamento de Biologia, Universidade Federal Rural de Pernambuco, R. Manuel de Medeiros s/n, Recife, PE 52171-900 Brazil; 10 Dipartimento di Scienze e Tecnologie Biologiche e Ambientali, University of Salento, Via Monteroni s/n, 73100 Lecce, Italy; 11 Reef Systems Research Group, Ecology Department, Leibniz Center for Tropical Marine Research, Fahrenheitstraße 6, 28359 Bremen, Germany; 12 TAXPO-Museu Nacional, Departamento de Invertebrados, Universidade Federal do Rio de Janeiro, Quinta da Boa Vista s/n, Rio de Janeiro, RJ 2090-040 Brazil; 13 Laboratório de Bentos, Instituto de Ciências Biológicas, Universidade de Brasília, Campus Darcy Ribeiro s/n, Brasília, DF 70910-900 Brazil; 14 Laboratório de Biologia Recifal, Instituto Oceanográfico, Universidade de São Paulo, Praça do Oceanográfico 191, Cidade Universitária, São Paulo, SP 05508-120 Brazil; 15 Center for Marine and Environmental Studies, University of the Virgin Islands, 2 John Brewers Bay, St. Thomas, 00802 US Virgin Islands

**Keywords:** Ocean sciences, Coral reefs, Geology

## Abstract

Large gaps in reef distribution may hinder the dispersal of marine organisms, interrupting processes vital to the maintenance of biodiversity. Here we show the presence and location of extensive reef habitats on the continental shelf between the Amazon Reef System (ARS) and the Eastern Brazilian Reef System (ERS), two reef complexes off eastern South America. Formations located 20–50 m deep include both biogenic and geogenic structures. The presence of diverse reef assemblages suggests the widespread occurrence of rocky substrates below 50 m. These habitats represent an expansion of both the ARS and ERS and the closure of the only remaining large-scale gap (~ 1000 km) among West Atlantic reef environments. This indicates that the SW Atlantic harbors a single, yet heterogeneous, reef system that stretches for about 4000 km, and thus, represents one of the largest semi-continuous tropical marine ecosystems in the world.

## Introduction

**Figure 1:**
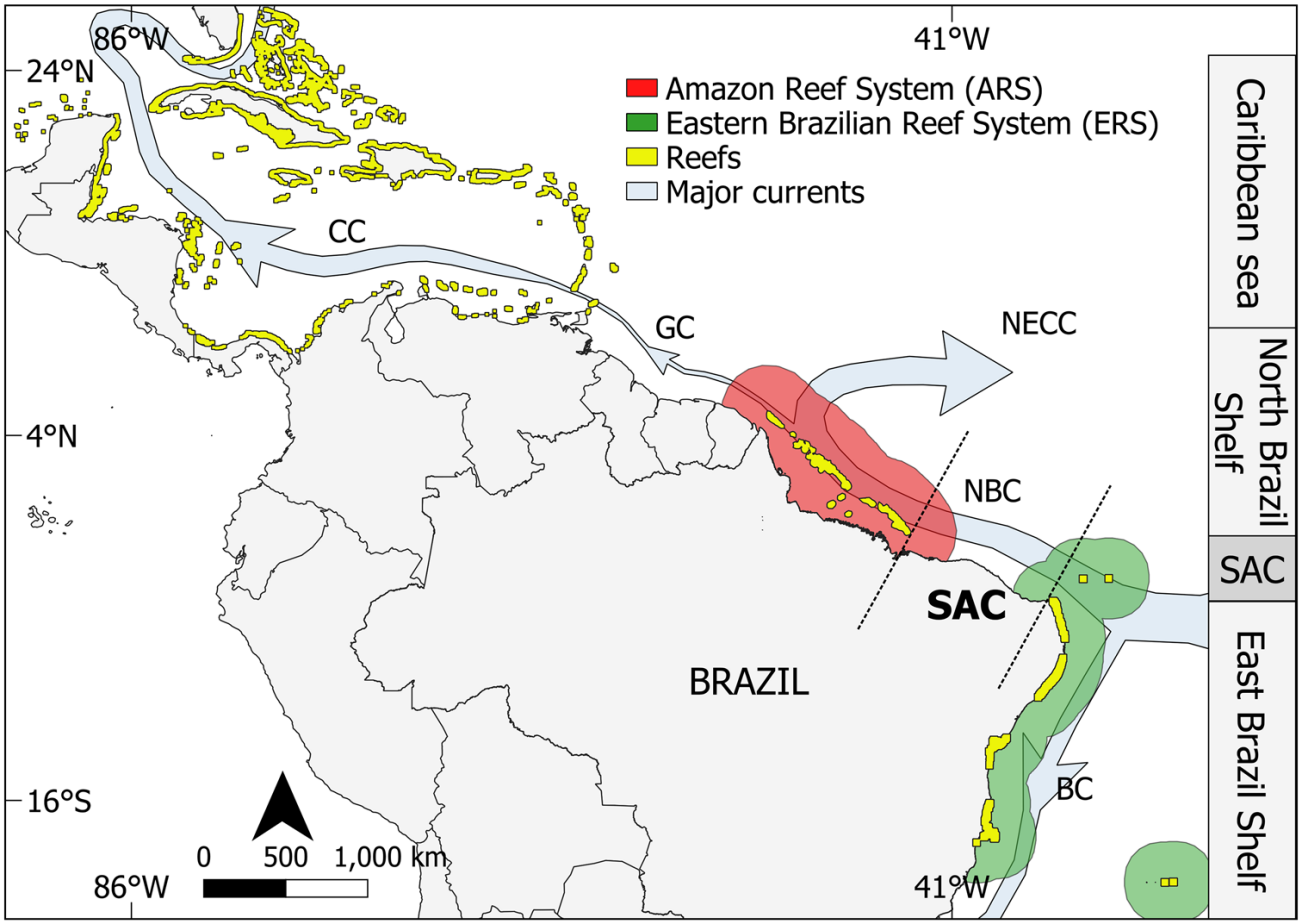
West coast of the Atlantic Ocean showing the countries of Central and South America, large marine ecosystems, major reef systems, and main ocean currents. Areas of reefs and reef systems are out of scale. Shallow reef distribution modified from^15^ and Amazon reefs based on^8^. *SAC* Brazilian semi-arid coast, *BC* Brazil current, *NBC* North Brazil current, *NECC* North equatorial countercurrent, *GC* Guiana current, *CC* Caribbean current. Map made using QGIS 3.22 (https://qgis.org/). Country areas retrieved from GADM (https://gadm.org/).

Large clusters of interconnected reefs extending for hundreds to thousands of square kilometers—referred to as reef systems^1^—are an important feature of tropical coasts. Along the South Atlantic Ocean two main reef systems have been recognized to date: the Eastern Brazilian Reef System (ERS), which comprises shallow-water (< 30 m depth) and mesophotic (30–150 m) reefs along Brazil's eastern continental shelf^2–4^; and the Amazon Reef System (ARS), encompassing mesophotic and rariphotic (30–220 m depth) ecosystems located on the Brazilian Equatorial Margin^5–10^ (Fig. 1). In both cases, reefs are associated with other habitats, such as rhodolith beds, sponge bottoms and seagrass meadows, and the reef systems are more properly a mosaic of interconnected benthic habitats^8,10,11^. Furthermore, the ARS may provide a bridge between the SW Atlantic and Caribbean, serving as an ecological corridor allowing the exchange of reef species between biogeographic provinces^7,12–14^.

Between the ARS and ERS (from coordinates 5° S 36° W to 2° S 44° W) is the Brazilian semi-arid coast (SAC)^16^. This area extends for about 1000 km in the SE-NW direction, and has distinct environmental conditions, such as a large interannual variability in rainfall, a predominance of small rivers, and a strong influence of wind on coastal landscapes^16^. Historically, the continental shelf along the SAC is considered poor in terms of biogenic reef occurrence^17–19^. The shallower sector of this continental shelf is subject to intense wind-driven longshore sediment drifts^16^, and large biogenic reefs are absent^20^. In nearshore areas, only fossil biogenic formations or small coral assemblages on intertidal rock outcrops have been reported, which are usually referred to as sandstone reefs^20^. However, the warm, clear, and oligotrophic waters make the deeper sectors of the SAC continental shelf suitable for the growth of calcifying organisms, and these areas do have a significant modern CaCO_3_ production^21–23^. Accordingly, the occurrence of biodiverse “coral banks” have been reported for these deeper sectors at least since the 1970s^19^, and some maps from that decade even mention deep-water “algal reefs”^24^. However, these reports were rather uncertain and mentioned structures were hardly ever described as reefs by subsequent authors, who mostly reported unconsolidated carbonate substrates such as rhodolith beds or carbonate sand and gravel^22,25,26^. Therefore the SAC is considered to be depauperate in the most recent reviews on Brazilian reefs^3,4,17,18,27,28^.

Nevertheless, in the past decade a growing number of studies have reported the occurrence of hard substrates in this region, located from the intertidal zone to tens of meters below the surface^29–34^. Biological and geological characterizations of these substrates are only beginning, but many sustain a diverse reef-associated biota (i.e., reef fishes and epilithic benthos), which play an important role in the economy and ecology of the region^32,35–37^. Furthermore, there are similarities between the SAC hard substrates and the biogenic reefs from the ARS and ERS^2,33,34,38^.

To date most of the studies along the SAC have been limited and it is not possible to delineate the extent and distribution of these hard-bottom environments, or to put them in a context among other Brazilian reef systems. A better understanding of the distribution of reefal habitats along the SAC is required to improve the uncertainty regarding connectivity between these tropical reef systems. Large gaps between suitable habitats could, for example, hinder the dispersal of marine organisms^39,40^. In this context, the North Brazil Current (NBC), which flows northwestward off shelf, has surface velocities around 60–100 cm s^−1^^41^ (Fig. 1). But wind-driven shelf currents are slower, with an average velocity of about 20 cm s^−1^^42^. At this speed, pelagic larvae would take around 60 days to cross the entire SAC, from the northern ERS tip to eastern ARS tip, which is longer than the planktonic larval duration of many reef species^43,44^. Accordingly, there seems to be a low demographic connectivity among populations of certain reef species along the equatorial SW Atlantic^45^. So rocky substrates along the SAC region may act as stepping-stones, aiding biological flux between ERS and ARS^45^. As such, an assessment of the types and locations of reef habitats along this area could help to clarify such a role.

Here we assess the occurrence and distribution of shallow-water and mesophotic hard-bottom habitats along the SAC continental shelf, by integrating new data with published and unpublished records. This integration of data provides evidence that ARS and ERS are spatially connected by numerous hard-bottom habitats distributed along the continental margin, and thus form a single extensive reef ecosystem extending from the southern Caribbean to the tropical southwestern Atlantic Ocean.

## Results

### Occurrence and distribution of Hard-bottom environments

**Figure 2:**
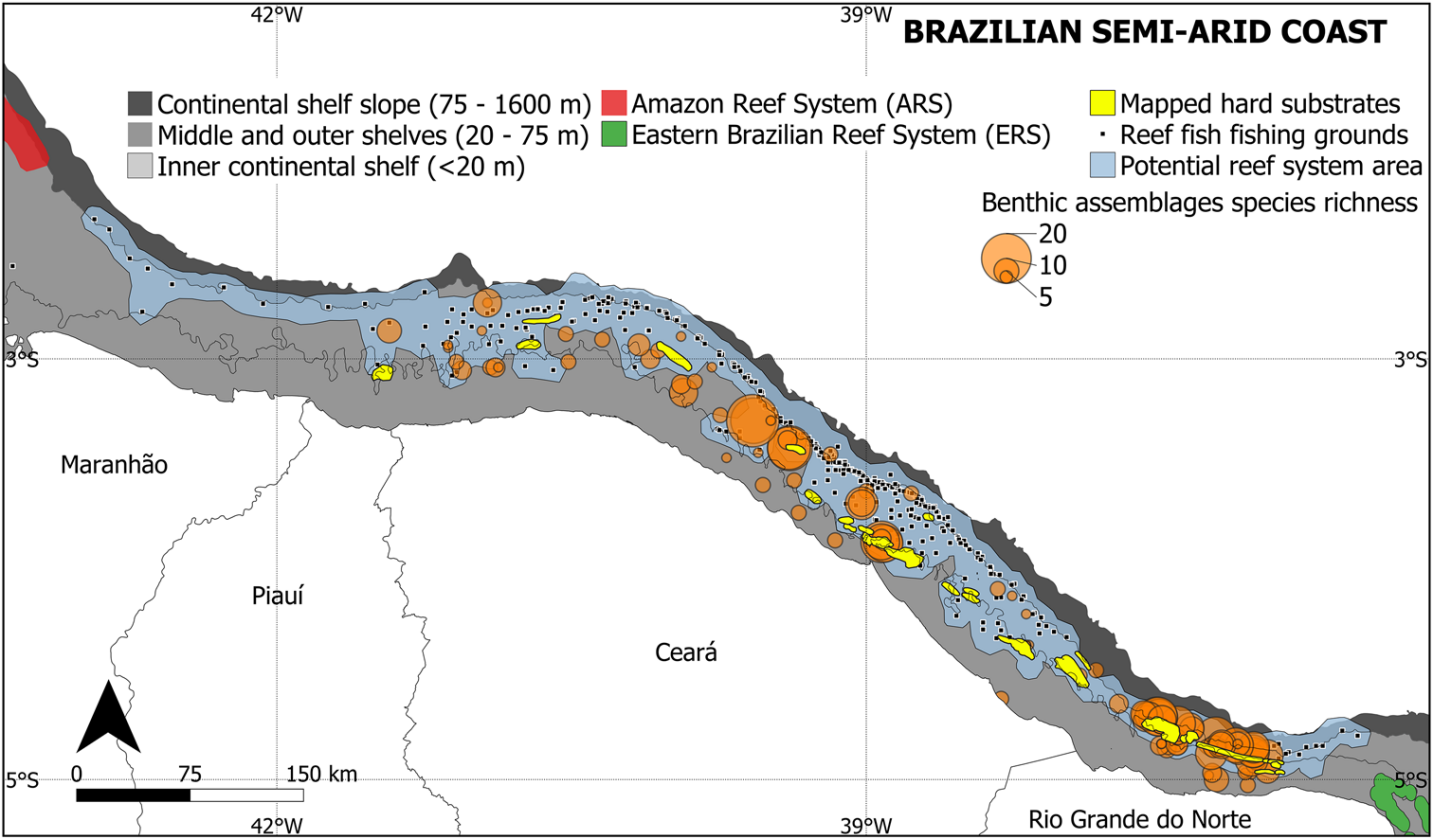
Distribution of euphotic and mesophotic hard-bottom habitats along the Brazilian semi-arid coast (Tropical SW Atlantic). Location data for benthic assemblages (including octocoral, scleractinian, and sponge grounds typically with reef fish assemblages) and fishing grounds obtained from the literature, scientific collections, and the monitoring of commercial fisheries (a list of data sources can be found in the supplementary materials S1). Mapped hard substrates depict known rocky-bottom environments, while benthic assemblages and fishing grounds indicate putative rocky substrates. Map made using QGIS 3.22 (https://qgis.org/). Bathymetric data retrieved from CPRM (https://www.cprm.gov.br/) and country area from GADM (https://gadm.org/).

There are records of hard substrates along most of the SAC continental margin. Eleven studies have reported a total of 192 sites of geo-referenced hard-bottom habitats, particularly between 20 and 50 m depth, including at least 59 named formations (Supplementary Table S1). Although the geological composition of these hard substrates was not determined in most of these studies, three articles reported CaCO_3_-cemented sandstones, most likely submerged beachrock, and one described a limestone covered by a well-developed coral assemblage (the Açu reefs), thus confirming the occurrence of biogenic reefs along the SAC (Supplementary Table S1). It is important to note that only primary studies are being reported here, and the nature of both the sandstone and limestone formations have been supported by subsequent studies^29–31,38,46–49^. Another two studies^32,36^ mentioned large coral assemblages on unspecified substrates, but only one^32^ quantified the benthic cover, reporting a dominance by seaweeds and sponges, with live corals and calcareous algae covering together 9.2% of the substrate. In the present study, many of these rocky structures could be identified and mapped by Landsat images and bathymetry. This mapping shows a semi-continuous ridge of consolidated substrates parallel to the coast extending along the entire SAC middle continental shelf (Fig. 2).

In addition to these confirmed hard-bottom substrates, there are abundant records of epilithic communities along the SAC continental shelf (Supplementary Table S1). The literature and collection surveys resulted in 193 geo-referenced records of benthic assemblages: 99 records of sponge bottoms, with up to 47 species (mean 5.6 ± 7.0); 17 records of coral assemblages (Hexacorallia and Octocorallia), with up to 19 species (mean 3.4 ± 5.0); and 77 records of macroalgae, with up to 8 species (mean 1.7 ± 1.2). Many of these biotic communities co-occur with known rocky substrates, but some diverse assemblages were also found far from currently known hard substrates (Fig. 2), particularly along the continental shelf break where depth and distance from the coast prevent confirmation by satellite images or conventional scuba diving.

Despite the lack of detailed scientific descriptions, the outer SAC continental shelf is intensely fished for reef-fish species (Fig. 2). Three articles report at least 129 geo-referenced fishing grounds used primarily to the capture of reef species such as snappers (Lutjanidae) (Supplementary Table S1). Additionally, during the fishing campaigns monitored in the present study (Supplementary Table S2), 68.1% ± 12.9% of the biomass and 51.3% ± 6.8% of the species richness was composed of primarily reef-associated fish. Snappers (*Lutjanus*), black groupers (*Mycteroperca bonaci*), nurse sharks (*Ginglymostoma cirratum*) and stingrays (*Hypanus*) were present in more than 90% of the fishing grounds and accounted for 60.8% of the total captured biomass. Their abundance, along with the diverse benthic assemblages, is a strong indication of the occurrence of consolidated reefal substrates along the SAC outer continental shelf.

### Ground-truthing

**Figure 3:**
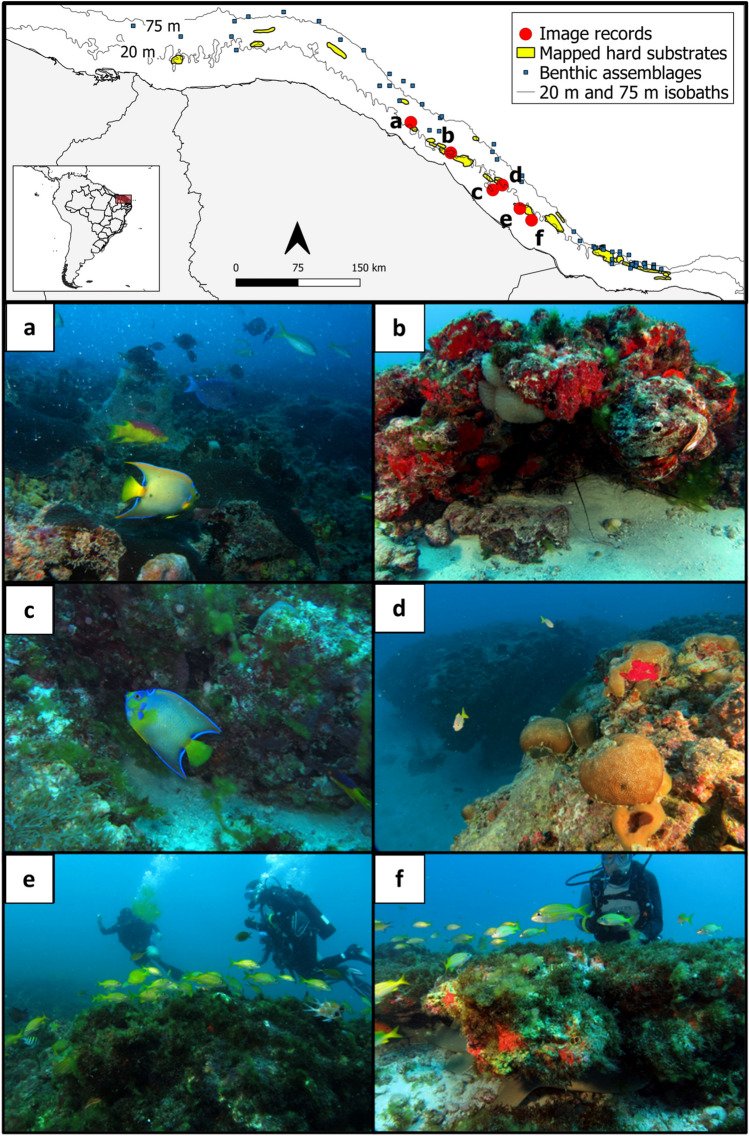
Reefal environments on the continental shelf off the Brazilian semi-arid coast. (**a**) “Manoel Salvador” reef (~ 30 m deep) covered by *Montastraea cavernosa*, (**b**) “Pedrinha” (~ 17 m) with sponges, ascidians, and calcareous algae, (**c**) reef fish (*Holacanthus ciliaris*) on the “Cabeço Seco” (~ 13 m) (**d**) “Canal do Uruaú” mesophotic reef (~ 34 m) with large *Siderastrea* spp. coral colonies, (**e**) “Pedra Grande da Majorlandia” (~ 16 m) covered by fleshy algae, and (**f**) “Pedra Preta” (~ 13 m) covered by fleshy and calcareous algae. Map made using QGIS 3.22 (https://qgis.org/). Bathymetric data retrieved from CPRM (https://www.cprm.gov.br/) and country area from GADM (https://gadm.org/).

Our ground-truthing of the reported rocky substrates using Scuba diving indicates that these habitats usually rise a few centimeters to a few meters above the surrounding sandy substrate, forming plateaus with positive relief (Fig. 2 and map in Fig. 3). All visited reefal formations sustain a diverse benthic community, usually with a cover of calcifying organisms, such as reef-building scleractinian corals (mostly *Montastraea cavernosa* and *Siderastrea* spp.) (Fig. 3a,d) and calcareous algae (Fig. 3f). Reef fish assemblages were also present. Habitats located at greater depths, such as the Manoel Salvador (Fig. 3a) and the Uruaú Channel (Fig. 3d), have significant live coral cover. Whereas shallower formations sustain a noticeable layer of calcareous algae (note, for example, the purplish color of the substrate in Fig. 3b,c). These observations indicate a considerable biological contribution to these structures.

**Figure 4:**
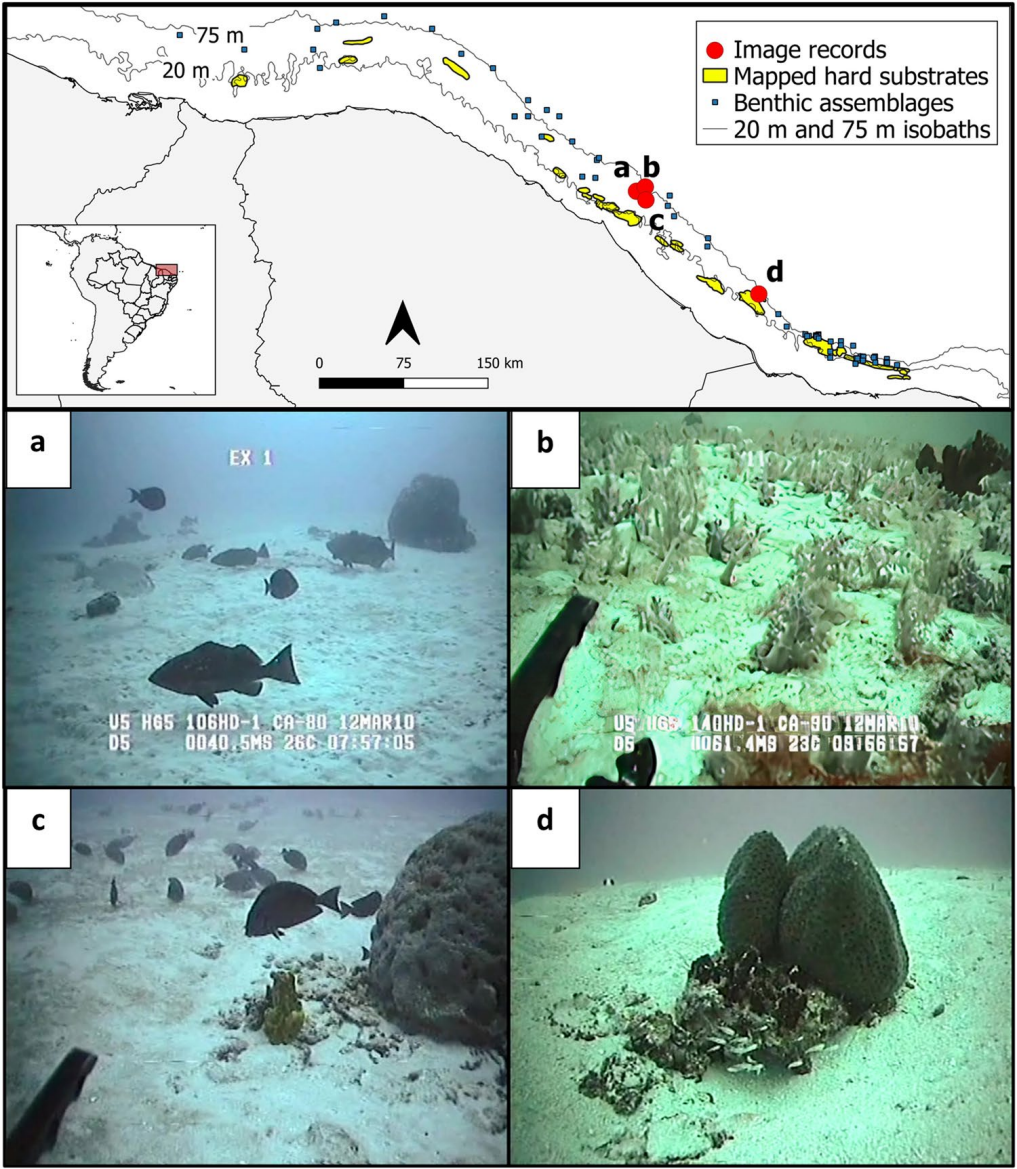
Putative rocky substrates on the outer continental shelf off the Brazilian semi-arid coast. Massive sponges and reef-associated fishes (families Acanthuridae, Lutjanidae, and Epinephelidae) observed by Remotely Operated Underwater Vehicle (ROV) suggesting the occurrence of widespread hard substrates at mesophotic depths. (**a**) Massive sponges (*Agelas dispar*) and reef fishes (*Lutjanus jocu*, *Mycteroperca* sp., *Epinephelus* sp. and *Acanthurus* sp.) at 41 m deep, (**b**) benthic assemblage dominated by octocorals (*Neospongodes atlantica*) apparently on a rocky substrate at 61 m, (**c**) a massive sponge (*A. dispar*), a columnar sponge (*Aplysina* sp.), and a school of *Acanthurus* sp. on a low relief rocky substrate covered by a sand veneer at 36 m, (**d**) Oval sponge (*Ircinia strobilina*) on a small rocky outcrop surrounded by soft sediments at 41 m. Map made using QGIS 3.22 (https://qgis.org/). Bathymetric data retrieved from CPRM (https://www.cprm.gov.br/) and country area from GADM (https://gadm.org/).

Ground-truthing of four areas along the outer continental shelf by ROV also revealed complex habitats (Supplementary Video S1). Apparently, there are rocky ledges that do not rise distinctively above the surrounding sea floor, and often appeared covered by a sediment layer (Fig. 4). Due to this sand cover, the consolidated nature of these habitats is better seen in video than in still images. Scattered rocks, epilithic sponges (e.g., *Xestospongia* cf. *muta*, *Agelas dispar*, *Aplysina fulva*, *Aplysina lacunosa*, and *Ircinia strobilina*) and soft- and hard-bottom dwelling octocorals (such as *Neospongodes atlantica*) also appeared in these areas (Fig. 4). They also sustained typically reef-associated fish, including *Acanthurus* sp. (Acanthuridae), *Bodianus* sp. and *Halichoeres* sp. (Labridae), *Holocentrus adscensionis* (Holocentridae), *Lutjanus jocu* (Lutjanidae), *Mycteroperca* sp. and *Epinephelus* sp. (Epinephelidae) (Fig. 4a–d).

Due to their depths, the nature of these outer shelf habitats cannot be confirmed in the present study, therefore they are referred to as putative rocky substrates hereafter. Despite this uncertainty, confirmed and putative hard-bottom habitats occur relatively close to each other, forming a single reefal seascape that extends about 34,000 km^2^, and which is clearly aligned with ARS (to the west), possibly providing a bridge to ERS (to the east) along the equatorial margin of the SW Atlantic Ocean (Fig. 2).

### Relationships among south Atlantic reef systems

**Figure 5:**
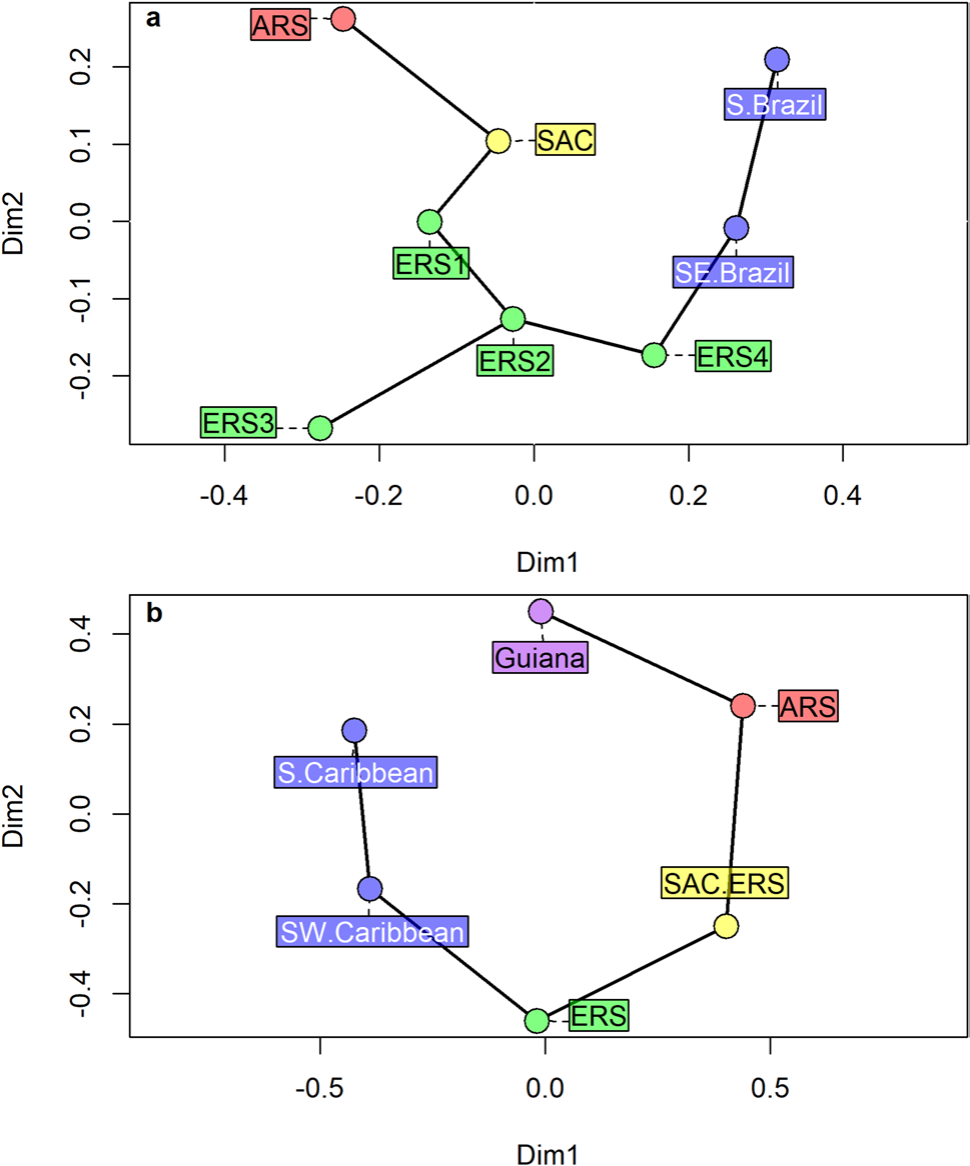
Ordination analyses (Sammon mapping) of South Atlantic reef systems and biogeographic regions based on the Sørensen dissimilarities among their marine biotas. (**a**) Analysis on a dataset with 2412 reef species, published by Ref.^51^, (**b**) analysis on a dataset with 8375 marine species, published by Ref.^50^. A minimum spanning tree was superimposed to the ordination graph in order to highlight putative connection pathways among regions^52,53^. Note that SAC is between ARS and ERS in both cases. *ARS* Amazon reef system, *SAC* Brazilian semi-arid coast reef system, *ERS* Eastern Brazilian reef system.

The role of SAC formations as stepping-stones between ARS and ERS is supported by biogeographic patterns. The ordination analyses of two large datasets of marine species^50,51^ suggests a direct relationship between the ecological dissimilarities (in terms of species composition) and the geographic distances of ARS, SAC, ERS and other South American biogeographic regions (Fig. 5). Moreover, the superimposition of a minimum spanning tree on the ordination analyses^52,53^ provides an indication that biogeographic links between ARS and ERS consistently pass-through SAC (Fig. 5). This same connection pattern is observed both for the entire dataset, and for the majority (64%) of individual high-rank taxonomic groups in each dataset (Supplementary Figs. S1 and S2).

These connections among ARS, SAC and ERS indicate that these reef systems can be regarded as a single entity across the South Atlantic Ocean, at least at the broader scales of analysis. However, each reef system (or subsystem) has particularities that should not be disregarded in ecological studies (Table 1). Overall, mesophotic shelf-edge reefs seem to occur along most of the Brazilian continental margin, but as formations become shallower, their number, variety and biodiversity increase from north to south.

**Table 1:** Summary description of the main sectors comprising the South American Reef System.

	South American reef system		
Subsystems	Amazon Reef System^5–10,14,54^	Semiarid Coast Reef System^20,29–33,35,36,38,46–49^	Eastern Brazilian Reef System^3,4,17–19,55^
Extension	~ 1000 km	~ 1000 km	~ 2000 km
Geological settings	Amazonas, Pará-Maranhão, and Barreirinhas sedimentary basins	Barreirinhas, Ceará, and Potiguar sedimentary basins	Eastern Brazilian margin sedimentary basins (between Pernambuco-Paraiba, and Santos basins)
Types of reef environments	Non-framework-building communities and shelf edge biogenic reefs^a^	Non-framework-building communities, submerged patch reefs, and shelf edge biogenic reefs. Relict fringing reefs may be present	Non-framework-building communities, fringing reefs, patches, banks, pinnacles (“chapeirões”), and shelf edge biogenic reefs. Highest coral richness in the SW Atlantic coast
Location of reef environments	Middle and outer continental shelf and shelf break, with reef tops located hundreds of meters below the water^a^	Inner, middle, and outer continental shelf to the shelf break, with reef tops located from tens to hundreds of meters below the water	Inner, middle, and outer continental shelves, from the shore to the shelf break, with reef tops located from the surface to tens of meters below the water
Substrate of reef environments	Topographic heights left by erosion of Pleistocene sandstones and carbonates	Cemented terraces, beachrock lines and topographic heights left by erosion of Pleistocene carbonates	Ancient crystalline and sedimentary rocks, cemented terraces, beachrocks lines and topographic heights left by erosion of Pleistocene carbonates
Size of reef framework	None to a few centimeters^a^	None to a few meters	None to tens of meters
Main framework building and/or foundation species	Crustose coralline algae (major component), bryozoans, corals (particularly *Madracis decactis*), and serpulids	Reef-building massive corals, and coralline algae	Reef-building scleractinians and hydrocorals, coralline algae, and bryozoans

^a^The Manuel Luís Reefs are exceptions. These reefs are located at the border of the Amazon and the Semi-Arid Reef Systems but are generally considered to belong to the former. They are coral-rich formations, which rise steeply to the surface and reach shallow depths^54^.

## Discussion

The available data confirm that reefal seascapes associated with hard-bottoms are extensive and semi-continuous along 34.000 km^2^ of the middle-to-outer continental shelf of the SAC. This discovery of a spatial connection between ARS and ERS implies that this should be regarded as a single and extensive reef system, stretching for about 4000 km along the east coast of South America.

The mapping and physical characterization of this reef system should be improved in future studies. Due to the limited penetration of light into water, analyzes based on satellite data are normally restricted to underwater features located no more than 40 m deep^1,29^. Therefore, new reef areas along the SAC must be surveyed and validated, especially close to the shelf break. In addition, the shallower (< 10 m) and western sectors of the SAC continental shelf are generally more turbid^33^ and therefore more difficult to map with remote sensing techniques. The use of high spectral, spatial, and temporal resolution data, in addition to statistical methods and direct underwater assessments (via technical diving, ROVs and BRUVs) are essential to refine our initial estimates of the reef distribution.

The geological composition of SAC hard-bottom formations is uncertain, but, as we have shown here, include sandstones cemented by calcium carbonate (i.e. beachrocks)^30^ and limestones with well-developed coral communities (i.e., biogenic reefs)^46^. The only study that quantified the benthic cover on one of these formations^32^, a rocky plateau at 23 m depth, reported that live corals and calcareous algae covered 9.2% (4.6% each) of the substrate. This number is within the range commonly observed in Brazilian biogenic reefs^54–56^. However, maybe except for the mostly organic Açu reefs, it is generally not known how much these bioconstructors contribute to the framework of the SAC habitats. Therefore, ecosystems that develop on these hard substrates seem to range from deep and low-lying marginal biogenic reefs^28,57,58^ to non-framework building communities^58^, including coral grounds and carpets^59,60^ which can be regarded as an integral part of the reef system along the SAC. At the same time, the prevalence of biogenic versus geogenic formations should be reassessed throughout the entire South American reef system.

Outside the alignment of rocky-bottom habitats between 20 and 40 m deep, numerous localities along the SAC continental shelf support diverse epilithic biotas. For example, trawls made at mesophotic depths revealed a high species richness and biomass of reef benthic organisms, with biodiversity apparently increasing with depth^61^. The dominant taxon in these surveys was Porifera, which accounted for 90% of the total wet weight, especially due to the species *Agelas dispar*, *Ircinia strobilina*, *Leucascus* sp. and *Monanchora arbuscula*. Ascidians were the second most abundant group, with the species *Stomozoa gigantea* alone representing 5% of the total wet weight, followed by Cnidaria (3% of the biomass) and Bryozoa (2% of the biomass)^61^. Previous studies have shown that such assemblages are associated with the occurrence of geoforms, including reefs and associated habitats^62^. Additionally, similar biotic communities were found in the deeper zones of the eastern Brazilian continental shelf (along the ERS) and are also linked to the occurrence of mesophotic reef habitats^63^. Therefore, the presence of these assemblages is a strong indication of the occurrence of yet unmapped hard-bottom environments along the SAC, especially in the outer continental shelf.

Habitats deeper than 40 m explored using ROV data show a relatively flat seascape and hard substrates can only be inferred from the presence of epilithic biotas. Such flat seascapes are not typical of reef environments, which, by definition, have three-dimensional profiles. Nevertheless, ROV data is localized, and sandy and rocky flats are a known feature of reefs along the SAC^32,38,46^. In fact many formations along the ARS^8,10^ and ERS^64^ are flat and largely covered by marine sediment. Consequently, these relatively flat habitats seem to be another important component of the South American reef system, particularly at mesophotic depths^10,31,64^. Due to their apparent ubiquity across ARS, SAC and ERS, these mesophotic environments may form an extensive ecological corridor along most of the SW Atlantic coast.

Therefore, at a broad spatial scale, the SAC continental shelf harbors an extensive network of different types of consolidated substrates, which form a complex and heterogeneous reef system. Furthermore, much like the ARS and ERS^2^, this reef system forms a seascape with other habitats, such as rhodolith beds, seagrass and seaweed meadows, marine animal forests, and unconsolidated sediment deposits^23,65^. Data on the structure and functioning of these ecosystems is scarce, but the few available comparisons, both from within and outside the SAC, indicate important differences in terms of species composition and ecological dynamics^11,66^. The recognition of these particularities is essential for the adequate management and conservation of these habitats. In parallel, the SAC reef system and neighboring habitats are all equally under the energetic oceanographic conditions across the equatorial Brazilian shelf^26,33,67^. As such, some of these environments may even be subject to seasonal cycles of burial and exposure, due to the transport of unconsolidated sediments and possible migration of subaqueous sand ripples and dunes^68,69^. This may explain why some of the hard-bottom habitats are dominated by few stress-tolerant and weedy reef-building corals adapted to sedimentation and moderate turbidity, such as *M. cavernosa* and *Siderastrea* spp.^2,27,32,36,60^.

Due to their large spatial and depth distribution, the hard-bottom formations along the SAC connect ARS and ERS. Together, these reef systems may also link the Caribbean and Brazilian biogeographic provinces. Hence, recognizing the presence and extension of the SAC hard bottoms implies closing the only remaining broad scale gap among Western Atlantic reef habitats. This physical connectivity is mediated by the shelf currents and the NBC, both of which flow primarily from east to west (Fig. 1). However, the NBC also curves back to the east (reflects) between July and December around 6–8° N (as shown in Fig. 1) shedding eddies and rings that may flow eastward, possibly helping to interconnect the reef environments^70,71^. In this scenario, the SAC hard substrates effectively constitute stepping-stones between ARS and ERS, providing adequate substrates for at least some species, aiding larval dispersion, ontogenetic migration, and small-scale movements of adult animals, which are key processes connecting Western Atlantic reef systems^45,72,73^. It is noteworthy that both ARS and SAC reefs are typically composed of subsets of the Tropical Atlantic biota, as their environmental conditions seem to be challenging for many coral reef species confined to either the ERS or Caribbean. Nevertheless, the spatial connection provided by the SAC may help to explain the biogeographical affinities observed in previous studies among SW Atlantic reef species, including sponges^74^, corals^75^, anemones^76^, gastropods^77^, and reef fish^78^. Furthermore, a multi-taxa study with reef organisms reports a relatively low beta diversity and high nestedness among Brazilian biogeographical provinces, suggesting considerable interconnectivity along the South American reef system^51^.

The connectivity between ARS and ERS provided by the SAC may affect the resilience of the SW Atlantic reefs against environmental and anthropogenic stressors^45^. Such human pressures are threatening the biodiversity of both shallow and mesophotic habitats and, thus, urgently require conservation and mitigation actions^79^. To be successful, such actions need to consider the biogeography and dynamics of metapopulations among SW Atlantic reefs, especially given the wide spatial distribution of such habitats^75,78^. Further study is urgently required to characterize the ecological dynamics of these tropical reef habitats at multiple spatial and temporal scales in order to sustainably maintain the numerous ecosystem services provided.

## Methods

Data on the occurrence of submerged hard-bottom substrates in this equatorial area was compiled from published and unpublished literature. Three types of reef-related data were compiled: (a) location of known hard-bottom habitats—i.e. rocky substrate topologically distinct from the surrounding sediment deposits, (b) occurrence of sponge, scleractinian or octocoral assemblages typical of hard-bottom habitats recorded by trawling or other indirect methods, and (c) coordinates of fishing grounds used to catch reef fishes, whose bottoms had not been characterized, but which may include reefs, marine animal forests, and rhodolith beds. The literature survey included publications in English and Portuguese, but only primary data sources were considered (i.e., only studies mentioning the coordinates of a particular location for the first time were included, and subsequent papers were disregarded). A relevant proportion of the data was only available through gray literature, so we focused on institutional repositories of Brazilian universities (*Universidade Federal do Ceará, Universidade Estadual do Ceará, Universidade Federal do Rio Grande do Norte*, and *Universidade Federal de Pernambuco*), governmental agencies (*Portal de Periódicos da Coordenação de Aperfeiçoamento de Pessoal de Nível Superior*), and all-purpose search engines (Google Scholar, Scielo, Science Direct, and Web of Science). Search terms included combinations of “reef”, “beachrock”, “continental shelf”, “Northeast Brazil”, “fisheries”, “fishing grounds”, “corals”, “octocorals”, and “sponges”, with their respective Portuguese translations (a list of the data sources is available as Supplementary Table S1).

This literature survey was complemented by new information produced during this study. Surveys of scientific collections’ databases (*Universidade Federal do Ceará, Universidade Federal de Pernambuco, Museu Nacional do Rio de Janeiro/Universidade Federal do Rio de Janeiro*, and Smithsonian National Museum of Natural History) were performed to assess the occurrence of sponges and corals (octocorals and scleractinians) along the SAC continental shelf, particularly near the shelf break (Supplementary Table S1). The location of fishing grounds was supplemented by data from a commercial fishing operation involving 35 fishing campaigns using bottom longlines along the SAC outer shelf (Supplementary Table S2). These commercial data were used to assess the occurrence and abundance of reef fish species (identified as such following^78^) closer to the shelf break. The fishing campaigns targeted exclusively mesopredators and predators, particularly of the Lutjanidae and Epinephelidae families, and elasmobranchs. Therefore, these data do not represent the entire fish diversity on the SAC outer continental shelf. Nevertheless, the eventual occurrence of typical reef-fish assemblages should reflect the distribution of hard-bottom environments^63^, thus meeting the objectives of the present study.

Based on these published and unpublished data, submerged rocky substrates were mapped using satellite imagery and bathymetric data. Landsat 8 (OLI sensor) and Landsat 5 (TM sensor) digital images were processed (morphological convolution filter, directional filters, mask, and color and band rendering) through bands 1 and 2 (0.45–0.52 µm), and RGB compositions (4-3-2 and 4-2-1) were produced to highlight the occurrence and distribution of hard substrates. Bathymetric contours were created to corroborate and complement satellite information, using bathymetric data from the Brazilian Navy (DHN), retrieved form board pages (no. 500, 600, and 700) and nautical charts (no. 21,700, 21,800, and 21,900). Owing to the clear oceanographic conditions along most of the tropical Brazilian coast, satellite data can be used effectively to map submerged reefs and other hard-bottom habitats^29^. However, due to limited light penetration in water, such analyses are normally restricted to underwater features located shallower than 40 m deep. Additionally, the shallower (< 10 m) and western sectors of the SAC continental shelf had higher sediment inputs and resuspension^29^, and thus were more difficult to map with satellite images.

Ground-truthing of compiled data was performed for selected reef formations by Scuba diving and Remotely Operated Underwater Vehicle (ROV) assessments (Supplementary Video S1). In both cases, punctual surveys were performed over the selected formations and underwater images were taken to describe the reef habitats. Due to the large extension of the SAC continental shelf, ground-truthing surveys focused on a representative set of habitats. Due to the rough sea conditions in this area (e.g., intense wind speed and seasonal swell waves), this selection was based primarily on reef accessibility (depth and distance from the coast). Data on the location and nature of hard-bottom habitats, as well as species occurrence records, were integrated into a Geographic Information System (GIS) to evaluate the distribution of the reef habitats along the SAC continental shelf and estimate their positions and distances relative to ARS and ERS.

To investigate possible interconnections among ARS, SAC and ERS we re-analyzed two recently published datasets on the distribution of marine species along the tropical SW Atlantic coast^50,51^. The first dataset contained literature data on 2412 typically reef species from eight coastal sectors along the Brazilian coast^51^. The second dataset included records taken from the Ocean Biodiversity Information System (OBIS) for 8375 species (reefal and non-reefal) distributed across six marine ecoregions encompassing the Brazilian, Guianan, and Caribbean coasts^50^. Both datasets were originally used to identify and describe connections among marine biogeographical regions along their studied coastlines, mainly through cluster analysis and the construction of dendrograms. In the present study, to highlight the interconnections among reef systems, we opted for an ordination analysis. A Sammon mapping^80^, which is similar to a Multidimensional Scaling putting emphasis on the neighborhood of points, was used to visualize the data. Additionally, we have superimposed a minimum spanning tree, constructed with Prim’s algorithm^81^, on the Sammon mapping to aid the visualization of connections. Variations of minimum spanning trees have been used in biogeography to investigate putative historical pathways of connection among marine areas^52,53^. Considering the mostly unidirectional flow of oceanic and shelf currents along ERS, SAC and ARS the minimum spanning tree should also be able to reflect the interconnection among reef systems in a parsimonious way. The ordination analyses and the algorithm for the minimum spanning trees were executed in R 4.1.2, with packages vegan and MASS.

## Supplementary Information

Supplementary Information 1.

Supplementary Information 2.

Supplementary Information 3.

Supplementary Information 4.

## Data Availability

All data needed to evaluate the conclusions of this paper are presented in the paper and/or the Supplementary Materials. A list of geographic coordinates (lat/lon), additional images, and video shootings of the studied habitats are available from the corresponding authors upon reasonable request.
