# Supplementary material for: Interconnected marine habitats form a single continental-scale reef system in South America
Source: Sci Rep. 2022 Oct 17;12:17359. doi: 10.1038/s41598-022-21341-x (PMC9576765; doi:10.1038/s41598-022-21341-x)
Supplement: Supplementary file 1 — Supplementary Information 1. [file 41598_2022_21341_MOESM1_ESM.pdf]

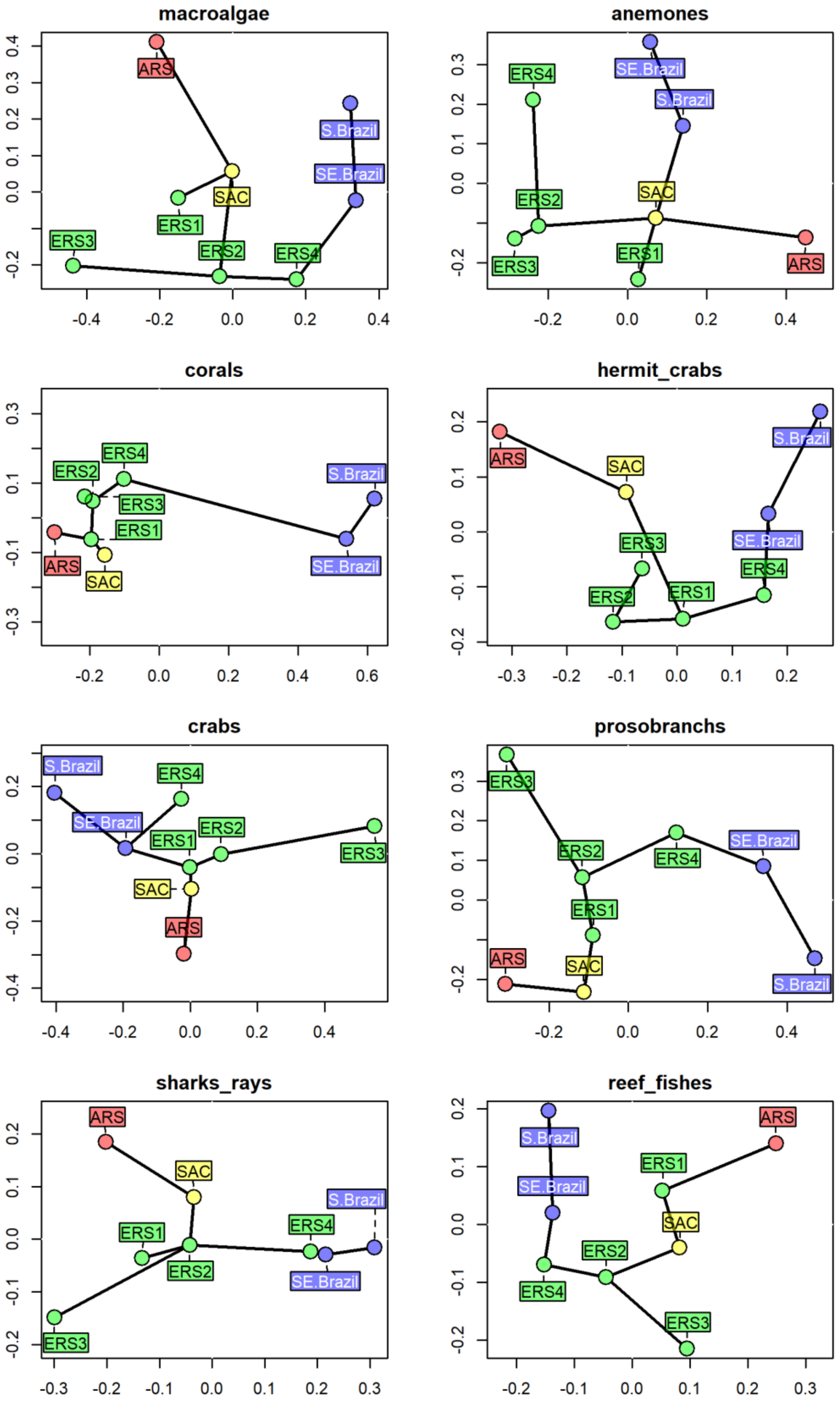

Fig. S1. Ordination analyses (Sammon mapping) of South Atlantic reef systems and biogeographic regions based on the Sørensen dissimilarities among their marine biotas. Analysis on a dataset with 2412 reef species, published by [47]. A minimum spanning tree was superimposed to the ordination graph in order to highlight putative connection pathways among regions [48,49]. Note that SAC is between ARS and ERS in the majority of cases. ARS = Amazon Reef System, SAC = Brazilian Semi-Arid Coast reef system, ERS = Eastern Brazilian reef system.

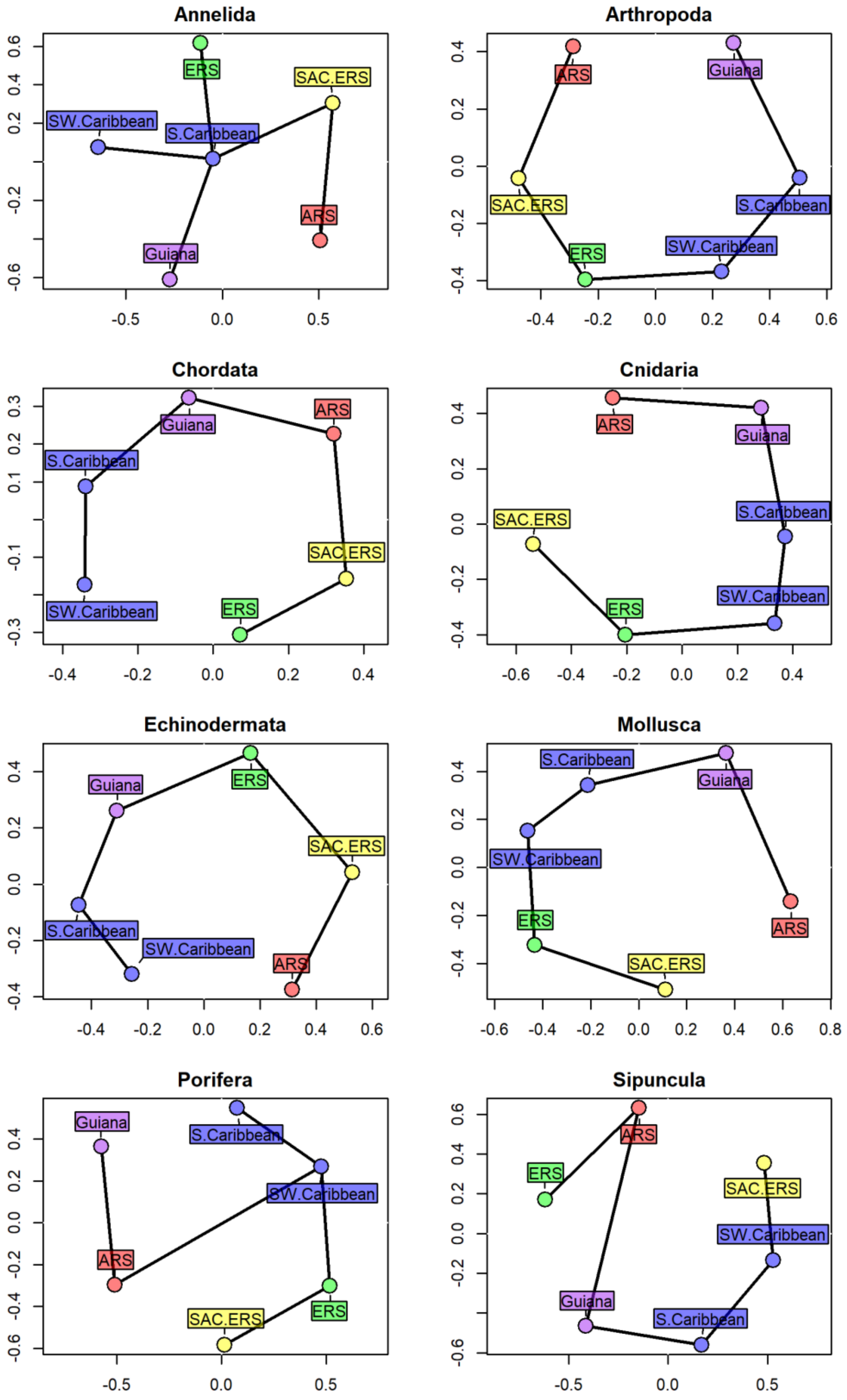

Fig. S2. Ordination analyses (Sammon mapping) of South Atlantic reef systems and biogeographic regions based on the Sørensen dissimilarities among their marine biotas. Analysis on a dataset with 8375 marine species, published by [46]. A minimum spanning tree was superimposed to the ordination graph in order to highlight putative connection pathways among regions [48,49]. Note that SAC is between ARS and ERS in half of the cases. ARS = Amazon Reef System, SAC = Brazilian Semi-Arid Coast reef system, ERS = Eastern Brazilian reef system.
