# Supplementary material for: Interconnected marine habitats form a single continental-scale reef system in South America
Source: Sci Rep. 2022 Oct 17;12:17359. doi: 10.1038/s41598-022-21341-x (PMC9576765; doi:10.1038/s41598-022-21341-x)
Supplement: Supplementary file 2 — Supplementary Information 2. [file 41598_2022_21341_MOESM2_ESM.docx]

**Supplementary Table S1.**  List of georeferenced localities with either known rocky substrates (i.e. Reef), reef-fish fishing grounds (i.e. fisheries) or epilithic benthic assemblages (i.e. Porifera, Coral and Algae) along the Brazilian semi-arid coast.

| **Locality** | **Type** | **Longitude** | **Latitude** | **Geology** | **References** |
| --- | --- | --- | --- | --- | --- |
| 1 | Reef | -37.369 | -4.481 | - | 1 |
| 2 | Reef | -37.374 | -4.48 | - | 1 |
| 3 | Reef | -37.392 | -4.481 | - | 1 |
| 4 | Reef | -37.417 | -4.484 | - | 1 |
| 5 | Reef | -37.424 | -4.482 | - | 1 |
| 6 | Reef | -37.432 | -4.482 | - | 1 |
| 7 | Reef | -37.439 | -4.483 | - | 1 |
| 8 | Reef | -37.345 | -4.481 | - | 1 |
| 9 | Reef | -37.335 | -4.484 | - | 1 |
| 10 | Reef | -37.325 | -4.484 | - | 1 |
| 11 | Reef | -37.321 | -4.495 | - | 1 |
| 12 | Reef | -37.337 | -4.44 | - | 1 |
| 13 | Reef | -37.326 | -4.439 | - | 1 |
| 14 | Reef | -37.314 | -4.439 | - | 1 |
| 15 | Reef | -37.318 | -4.451 | - | 1 |
| 16 | Reef | -37.318 | -4.461 | - | 1 |
| 17 | Reef | -37.317 | -4.47 | - | 1 |
| 18 | Reef | -37.315 | -4.482 | - | 1 |
| 19 | Reef | -37.308 | -4.496 | - | 1 |
| 20 | Reef | -37.3 | -4.507 | - | 1 |
| 21 | Reef | -37.316 | -4.504 | - | 1 |
| 22 | Reef | -37.329 | -4.474 | - | 1 |
| 23 | Reef | -37.32 | -4.482 | - | 1 |
| 24 | Reef | -37.409 | -4.434 | - | 1 |
| 25 | Reef | -37.383 | -4.433 | - | 1 |
| 26 | Reef | -37.386 | -4.424 | - | 1 |
| 27 | Reef | -37.399 | -4.422 | - | 1 |
| 28 | Reef | -37.402 | -4.416 | - | 1 |
| 29 | Reef | -37.38 | -4.409 | - | 1 |
| 30 | Reef | -37.369 | -4.415 | - | 1 |
| 31 | Reef | -37.355 | -4.413 | - | 1 |
| 32 | Reef | -37.34 | -4.418 | - | 1 |
| 33 | Reef | -37.327 | -4.417 | - | 1 |
| 34 | Reef | -37.31 | -4.421 | - | 1 |
| 35 | Reef | -37.316 | -4.408 | - | 1 |
| 36 | Reef | -37.16 | -4.422 | - | 1 |
| 37 | Reef | -37.294 | -4.375 | - | 1 |
| 38 | Reef | -37.226 | -4.376 | - | 1 |
| 39 | Reef | -37.25 | -4.384 | - | 1 |
| 40 | Reef | -37.312 | -4.365 | - | 1 |
| 41 | Reef | -37.287 | -4.319 | - | 1 |
| 42 | Reef | -37.274 | -4.385 | - | 1 |
| 43 | Reef | -37.25 | -4.398 | - | 1 |
| 44 | Reef | -37.228 | -4.415 | - | 1 |
| 45 | Reef | -37.211 | -4.422 | - | 1 |
| 46 | Reef | -37.2 | -4.428 | - | 1 |
| 47 | Reef | -37.174 | -4.434 | - | 1 |
| 48 | Reef | -37.299 | -4.308 | - | 1 |
| 49 | Reef | -36.213 | -4.882 | Beachrock | 2 |
| 50 | Reef | -36.082 | -4.898 | Beachrock | 2 |
| 51 | Reef | -36.234 | -4.878 | Beachrock | 2 |
| 52 | Reef | -36.234 | -4.878 | Beachrock | 2 |
| 53 | Reef | -36.234 | -4.878 | Beachrock | 2 |
| 54 | Reef | -36.233 | -4.878 | Beachrock | 2 |
| 55 | Reef | -36.233 | -4.878 | Beachrock | 2 |
| 56 | Reef | -36.233 | -4.878 | Beachrock | 2 |
| 57 | Reef | -36.233 | -4.878 | Beachrock | 2 |
| 58 | Reef | -36.21 | -4.882 | Beachrock | 2 |
| 59 | Reef | -36.452 | -4.848 | Beachrock | 2 |
| 60 | Reef | -36.447 | -4.85 | Beachrock | 2 |
| 61 | Reef | -38.408 | -3.569 | - | 3 |
| 62 | Reef | -38.391 | -3.598 | - | 3 |
| 63 | Reef | -38.387 | -3.579 | - | 3 |
| 64 | Reef | -38.446 | -3.471 | - | 3 |
| 65 | Reef | -38.27 | -3.546 | - | 3 |
| 66 | Reef | -38.456 | -3.44 | - | 3 |
| 67 | Reef | -38.837 | -3.264 | - | 3 |
| 68 | Reef | -38.431 | -3.575 | - | 3 |
| 69 | Reef | -38.381 | -3.573 | - | 3 |
| 70 | Reef | -38.383 | -3.579 | - | 3 |
| 71 | Reef | -38.403 | -3.595 | - | 3 |
| 72 | Reef | -37.248 | -4.343 | - | 3 |
| 73 | Reef | -38.383 | -3.579 | - | 4 |
| 74 | Reef | -38.408 | -3.569 | - | 4 |
| 75 | Reef | -36.817 | -4.7 | - | 5 |
| 76 | Reef | -36.461 | -4.906 | - | 5 |
| 77 | Reef | -36.338 | -4.958 | - | 5 |
| 78 | Reef | -36.3 | -4.883 | - | 5 |
| 79 | Reef | -36.167 | -4.95 | - | 5 |
| 80 | Reef | -36.167 | -4.883 | - | 5 |
| 81 | Reef | -36.083 | -4.917 | - | 5 |
| 82 | Reef | -36.017 | -4.9 | - | 5 |
| 83 | Reef | -35.85 | -4.917 | - | 5 |
| 84 | Reef | -37.726 | -4.123 | - | 6 |
| 85 | Reef | -37.779 | -4.181 | - | 6 |
| 89 | Reef | -38.521 | -3.605 | Beachrock | 7 |
| 90 | Reef | -38.548 | -3.466 | Beachrock | 7 |
| 91 | Reef | -38.433 | -3.458 | Beachrock | 7 |
| 92 | Reef | -38.432 | -3.573 | Beachrock | 7 |
| 93 | Reef | -38.383 | -3.576 | Beachrock | 7 |
| 94 | Reef | -38.393 | -3.597 | Beachrock | 7 |
| 95 | Reef | -38.236 | -3.72 | Beachrock | 7 |
| 96 | Reef | -38.303 | -3.718 | Beachrock | 7 |
| 97 | Reef | -38.29 | -3.686 | Beachrock | 7 |
| 98 | Reef | -38.268 | -3.658 | Beachrock | 7 |
| 99 | Reef | -38.244 | -3.665 | Beachrock | 7 |
| 100 | Reef | -38.222 | -3.659 | Beachrock | 7 |
| 101 | Reef | -37.846 | -3.943 | Beachrock | 7 |
| 102 | Reef | -37.936 | -3.955 | Beachrock | 7 |
| 103 | Reef | -37.947 | -4.005 | Beachrock | 7 |
| 104 | Reef | -37.946 | -3.995 | Beachrock | 7 |
| 105 | Reef | -37.773 | -3.862 | Beachrock | 7 |
| 106 | Reef | -37.953 | -4.175 | Beachrock | 7 |
| 107 | Reef | -37.528 | -4.324 | Beachrock | 7 |
| 108 | Reef | -37.25 | -4.343 | Beachrock | 7 |
| 109 | Reef | -40.502 | -2.415 | Beachrock | 8 |
| 110 | Reef | -39.712 | -2.428 | Beachrock | 8 |
| 111 | Reef | -39.56 | -2.533 | Beachrock | 8 |
| 112 | Reef | -38.918 | -3.033 | Beachrock | 8 |
| 113 | Reef | -38.823 | -3.317 | Beachrock | 8 |
| 114 | Reef | -38.618 | -3.46 | Beachrock | 8 |
| 115 | Reef | -38.127 | -3.436 | Beachrock | 8 |
| 116 | Reef | -37.997 | -3.866 | Beachrock | 8 |
| 117 | Reef | -37.87 | -3.886 | Beachrock | 8 |
| 118 | Reef | -37.904 | -3.913 | Beachrock | 8 |
| 119 | Reef | -37.588 | -4.193 | Beachrock | 8 |
| 120 | Reef | -37.536 | -4.248 | Beachrock | 8 |
| 121 | Reef | -37.338 | -4.311 | Beachrock | 8 |
| 122 | Reef | -37.211 | -4.42 | Beachrock | 8 |
| 123 | Reef | -38.704 | -3.216 | - | 9 |
| 126 | Reef | -38.456 | -3.616 | - | 9 |
| 128 | Reef | -38.423 | -3.566 | - | 9 |
| 130 | Reef | -38.405 | -3.567 | - | 9 |
| 131 | Reef | -38.387 | -3.566 | - | 9 |
| 132 | Reef | -38.387 | -3.583 | - | 9 |
| 133 | Reef | -38.371 | -3.566 | - | 9 |
| 134 | Reef | -38.272 | -3.532 | - | 9 |
| 135 | Reef | -38.322 | -3.666 | - | 9 |
| 136 | Reef | -38.338 | -3.75 | - | 9 |
| 137 | Reef | -38.288 | -3.684 | - | 9 |
| 138 | Reef | -38.255 | -3.65 | - | 9 |
| 139 | Reef | -38.288 | -3.716 | - | 9 |
| 140 | Reef | -38.206 | -3.599 | - | 9 |
| 141 | Reef | -38.222 | -3.651 | - | 9 |
| 142 | Reef | -38.222 | -3.716 | - | 9 |
| 144 | Reef | -38.522 | -3.484 | - | 9 |
| 145 | Reef | -38.456 | -3.433 | - | 9 |
| 146 | Reef | -38.439 | -3.467 | - | 9 |
| 147 | Reef | -36.579 | -4.77 | Limestone | 10 |
| 148 | Reef | -36.587 | -4.758 | Limestone | 10 |
| 149 | Reef | -36.57 | -4.742 | Limestone | 10 |
| 150 | Reef | -36.579 | -4.732 | Limestone | 10 |
| 151 | Reef | -36.561 | -4.732 | Limestone | 10 |
| 152 | Reef | -36.549 | -4.746 | Limestone | 10 |
| 153 | Reef | -36.61 | -4.757 | Limestone | 10 |
| 154 | Reef | -36.611 | -4.768 | Limestone | 10 |
| 155 | Reef | -36.666 | -4.753 | Limestone | 10 |
| 156 | Reef | -36.669 | -4.732 | Limestone | 10 |
| 157 | Reef | -36.658 | -4.718 | Limestone | 10 |
| 158 | Reef | -36.666 | -4.691 | Limestone | 10 |
| 159 | Reef | -36.678 | -4.708 | Limestone | 10 |
| 160 | Reef | -36.693 | -4.737 | Limestone | 10 |
| 161 | Reef | -36.708 | -4.751 | Limestone | 10 |
| 162 | Reef | -36.706 | -4.718 | Limestone | 10 |
| 163 | Reef | -36.702 | -4.707 | Limestone | 10 |
| 164 | Reef | -36.687 | -4.679 | Limestone | 10 |
| 165 | Reef | -36.704 | -4.683 | Limestone | 10 |
| 166 | Reef | -36.723 | -4.707 | Limestone | 10 |
| 167 | Reef | -36.725 | -4.689 | Limestone | 10 |
| 168 | Reef | -36.719 | -4.671 | Limestone | 10 |
| 169 | Reef | -36.741 | -4.698 | Limestone | 10 |
| 170 | Reef | -36.745 | -4.683 | Limestone | 10 |
| 171 | Reef | -36.747 | -4.664 | Limestone | 10 |
| 172 | Reef | -36.746 | -4.649 | Limestone | 10 |
| 173 | Reef | -36.775 | -4.646 | Limestone | 10 |
| 174 | Reef | -36.775 | -4.683 | Limestone | 10 |
| 175 | Reef | -36.799 | -4.678 | Limestone | 10 |
| 176 | Reef | -36.828 | -4.661 | Limestone | 10 |
| 177 | Reef | -36.813 | -4.658 | Limestone | 10 |
| 178 | Reef | -36.791 | -4.663 | Limestone | 10 |
| 179 | Reef | -38.269 | -3.546 | - | 11 |
| 180 | Reef | -37.935 | -3.956 | - | 12 |
| 181 | Reef | -37.843 | -3.945 | - | 12 |
| 182 | Reef | -38.299 | -3.719 | - | 12 |
| 183 | Reef | -38.803 | -3.221 | - | 12 |
| 184 | Reef | -37.946 | -3.997 | - | 12 |
| 186 | Reef | -37.525 | -4.326 | - | 12 |
| 187 | Reef | -38.269 | -3.546 | - | 12 |
| 188 | Reef | -38.544 | -3.469 | - | 12 |
| 189 | Reef | -38.246 | -3.666 | - | 12 |
| 190 | Reef | -38.224 | -3.66 | - | 12 |
| 191 | Reef | -38.289 | -3.688 | - | 12 |
| 192 | Reef | -38.233 | -3.722 | - | 12 |
| 193 | Reef | -38.265 | -3.66 | - | 12 |
| 194 | Reef | -37.175 | -4.467 | - | 12 |
| 195 | Reef | -37.944 | -4.005 | - | 12 |
| 196 | Reef | -38.836 | -3.264 | - | 12 |
| 197 | Reef | -39.381 | -2.914 | - | 12 |
| 198 | Reef | -39.411 | -2.902 | - | 12 |
| 199 | Reef | -36.808 | -4.694 | - | 12 |
| 200 | Reef | -38.431 | -3.462 | - | 12 |
| 201 | Reef | -38.518 | -3.606 | - | 12 |
| 204 | Fishery | -37.455 | -4.53 | - | 13 |
| 205 | Fishery | -37.39 | -4.459 | - | 13 |
| 206 | Fishery | -37.439 | -4.483 | - | 13 |
| 207 | Fishery | -37.424 | -4.482 | - | 13 |
| 208 | Fishery | -37.374 | -4.48 | - | 13 |
| 209 | Fishery | -37.414 | -4.543 | - | 13 |
| 210 | Fishery | -37.392 | -4.481 | - | 13 |
| 211 | Fishery | -37.321 | -4.495 | - | 13 |
| 212 | Fishery | -37.386 | -4.554 | - | 13 |
| 213 | Fishery | -39.382 | -2.917 | - | 13 |
| 214 | Fishery | -39.372 | -2.924 | - | 13 |
| 215 | Fishery | -39.331 | -2.932 | - | 13 |
| 216 | Fishery | -39.25 | -2.972 | - | 13 |
| 217 | Fishery | -39.252 | -2.932 | - | 13 |
| 218 | Fishery | -38.198 | -3.333 | - | 14 |
| 219 | Fishery | -38.132 | -3.441 | - | 14 |
| 220 | Fishery | -37.94 | -3.595 | - | 14 |
| 221 | Fishery | -37.82 | -3.694 | - | 14 |
| 222 | Fishery | -37.913 | -3.806 | - | 14 |
| 223 | Fishery | -38.165 | -3.585 | - | 14 |
| 224 | Fishery | -38.109 | -3.566 | - | 14 |
| 225 | Fishery | -38.039 | -3.524 | - | 14 |
| 226 | Fishery | -38.086 | -3.655 | - | 14 |
| 227 | Fishery | -38.44 | -3.352 | - | 14 |
| 228 | Fishery | -38.43 | -3.268 | - | 14 |
| 229 | Fishery | -38.389 | -3.273 | - | 14 |
| 230 | Fishery | -38.356 | -3.189 | - | 14 |
| 231 | Fishery | -38.621 | -3.361 | - | 14 |
| 232 | Fishery | -38.58 | -3.319 | - | 14 |
| 233 | Fishery | -38.561 | -3.37 | - | 14 |
| 234 | Fishery | -38.449 | -3.454 | - | 14 |
| 235 | Fishery | -38.351 | -3.398 | - | 14 |
| 236 | Fishery | -38.351 | -3.445 | - | 14 |
| 237 | Fishery | -38.351 | -3.52 | - | 14 |
| 238 | Fishery | -38.3 | -3.431 | - | 14 |
| 239 | Fishery | -38.328 | -3.245 | - | 14 |
| 240 | Fishery | -38.328 | -3.301 | - | 14 |
| 241 | Fishery | -38.277 | -3.287 | - | 14 |
| 242 | Fishery | -38.314 | -3.347 | - | 14 |
| 243 | Fishery | -40.089 | -2.173 | - | 14 |
| 244 | Fishery | -40.038 | -2.22 | - | 14 |
| 245 | Fishery | -39.134 | -3.04 | - | 14 |
| 246 | Fishery | -39.008 | -2.872 | - | 14 |
| 247 | Fishery | -40.513 | -2.215 | - | 14 |
| 248 | Fishery | -40.41 | -2.215 | - | 14 |
| 249 | Fishery | -40.485 | -2.369 | - | 14 |
| 250 | Fishery | -40.168 | -2.234 | - | 14 |
| 251 | Fishery | -38.682 | -3.161 | - | 14 |
| 252 | Fishery | -38.635 | -3.184 | - | 14 |
| 253 | Fishery | -38.687 | -3.296 | - | 14 |
| 254 | Fishery | -38.533 | -3.226 | - | 14 |
| 255 | Fishery | -38.882 | -2.97 | - | 14 |
| 256 | Fishery | -38.752 | -3.091 | - | 14 |
| 257 | Fishery | -38.808 | -3.235 | - | 14 |
| 258 | Fishery | -38.729 | -3.221 | - | 14 |
| 259 | Fishery | -40.844 | -2.206 | - | 14 |
| 260 | Fishery | -40.844 | -2.308 | - | 14 |
| 261 | Fishery | -40.816 | -2.551 | - | 14 |
| 262 | Fishery | -40.927 | -2.639 | - | 14 |
| 263 | Fishery | -40.96 | -2.206 | - | 14 |
| 264 | Fishery | -41.011 | -2.411 | - | 14 |
| 265 | Fishery | -40.932 | -2.346 | - | 14 |
| 266 | Fishery | -40.895 | -2.28 | - | 14 |
| 267 | Fishery | -40.718 | -2.211 | - | 14 |
| 268 | Fishery | -40.662 | -2.304 | - | 14 |
| 269 | Fishery | -40.671 | -2.397 | - | 14 |
| 270 | Fishery | -40.578 | -2.318 | - | 14 |
| 271 | Fishery | -40.778 | -2.644 | - | 14 |
| 272 | Fishery | -40.648 | -2.676 | - | 14 |
| 273 | Fishery | -40.732 | -2.304 | - | 14 |
| 274 | Fishery | -40.75 | -2.229 | - | 14 |
| 275 | Fishery | -42.767 | -1.964 | - | 14 |
| 276 | Fishery | -42.623 | -2.057 | - | 14 |
| 277 | Fishery | -42.8 | -2.22 | - | 14 |
| 278 | Fishery | -42.316 | -2.075 | - | 14 |
| 279 | Fishery | -43.084 | -1.67 | - | 14 |
| 280 | Fishery | -42.996 | -1.731 | - | 14 |
| 281 | Fishery | -42.875 | -1.903 | - | 14 |
| 282 | Fishery | -41.333 | -2.285 | - | 14 |
| 283 | Fishery | -41.123 | -2.103 | - | 14 |
| 284 | Fishery | -41.118 | -2.308 | - | 14 |
| 285 | Fishery | -40.988 | -2.28 | - | 14 |
| 286 | Fishery | -42.083 | -2.173 | - | 14 |
| 287 | Fishery | -41.696 | -2.192 | - | 14 |
| 288 | Fishery | -41.477 | -2.173 | - | 14 |
| 289 | Fishery | -41.43 | -2.322 | - | 14 |
| 290 | Fishery | -36.027 | -4.94 | - | 14 |
| 291 | Fishery | -35.983 | -4.961 | - | 14 |
| 292 | Fishery | -36.087 | -4.933 | - | 14 |
| 293 | Fishery | -36.208 | -4.805 | - | 14 |
| 294 | Fishery | -36.115 | -4.805 | - | 14 |
| 295 | Fishery | -36.05 | -4.789 | - | 14 |
| 296 | Fishery | -35.992 | -4.838 | - | 14 |
| 297 | Fishery | -37.76 | -3.778 | - | 14 |
| 298 | Fishery | -37.699 | -3.918 | - | 14 |
| 299 | Fishery | -37.578 | -3.932 | - | 14 |
| 300 | Fishery | -36.262 | -4.798 | - | 14 |
| 301 | Fishery | -35.673 | -4.71 | - | 14 |
| 302 | Fishery | -35.582 | -4.74 | - | 14 |
| 303 | Fishery | -35.964 | -4.933 | - | 14 |
| 304 | Fishery | -36.057 | -4.91 | - | 14 |
| 305 | Fishery | -35.929 | -4.852 | - | 14 |
| 306 | Fishery | -35.887 | -4.808 | - | 14 |
| 307 | Fishery | -35.829 | -4.801 | - | 14 |
| 308 | Fishery | -35.789 | -4.801 | - | 14 |
| 309 | Fishery | -38.188 | -3.45 | - | 14 |
| 310 | Fishery | -38.142 | -3.492 | - | 14 |
| 311 | Fishery | -38.132 | -3.436 | - | 14 |
| 312 | Fishery | -38.17 | -3.655 | - | 14 |
| 313 | Fishery | -38.249 | -3.398 | - | 14 |
| 314 | Fishery | -38.286 | -3.515 | - | 14 |
| 315 | Fishery | -38.235 | -3.431 | - | 14 |
| 316 | Fishery | -37.476 | -4.081 | - | 15 |
| 318 | Fishery | -36.25 | -5.006 | - | 15 |
| 320 | Fishery | -38.314 | -3.543 | - | 15 |
| 321 | Fishery | -38.314 | -3.575 | - | 15 |
| 322 | Fishery | -38.244 | -3.594 | - | 15 |
| 323 | Fishery | -38.337 | -3.655 | - | 15 |
| 324 | Fishery | -38.44 | -3.459 | - | 15 |
| 325 | Fishery | -38.23 | -3.394 | - | 15 |
| 326 | Fishery | -38.202 | -3.356 | - | 15 |
| 327 | Fishery | -38.23 | -3.496 | - | 15 |
| 328 | Fishery | -38.03 | -4.032 | - | 15 |
| 329 | Fishery | -37.997 | -4.092 | - | 15 |
| 330 | Fishery | -37.965 | -4.027 | - | 15 |
| 331 | Fishery | -37.955 | -3.934 | - | 15 |
| 332 | Fishery | -38.179 | -3.729 | - | 15 |
| 333 | Fishery | -38.002 | -3.594 | - | 15 |
| 335 | Fishery | -38.067 | -3.99 | - | 15 |
| 336 | Fishery | -40.741 | -2.413 | - | 15 |
| 337 | Fishery | -40.536 | -2.413 | - | 15 |
| 338 | Fishery | -40.527 | -2.544 | - | 15 |
| 339 | Fishery | -40.359 | -2.567 | - | 15 |
| 340 | Fishery | -41.403 | -2.534 | - | 15 |
| 341 | Fishery | -41.132 | -2.422 | - | 15 |
| 342 | Fishery | -40.96 | -2.599 | - | 15 |
| 343 | Fishery | -40.755 | -2.618 | - | 15 |
| 344 | Fishery | -40.778 | -2.586 | - | 15 |
| 345 | Fishery | -38.463 | -3.287 | - | 15 |
| 346 | Fishery | -38.584 | -3.459 | - | 15 |
| 347 | Fishery | -40.657 | -2.679 | - | 15 |
| 349 | Fishery | -40.834 | -2.711 | - | 15 |
| 350 | Fishery | -40.769 | -2.651 | - | 15 |
| 351 | Fishery | -36.143 | -4.861 | - | 15 |
| 352 | Fishery | -36.078 | -4.801 | - | 15 |
| 353 | Fishery | -36.05 | -4.875 | - | 15 |
| 354 | Fishery | -36.008 | -4.847 | - | 15 |
| 355 | Fishery | -37.699 | -4.113 | - | 15 |
| 356 | Fishery | -37.648 | -4.155 | - | 15 |
| 357 | Fishery | -37.727 | -4.16 | - | 15 |
| 358 | Fishery | -37.471 | -4.132 | - | 15 |
| 359 | Fishery | -37.727 | -3.92 | - | 15 |
| 360 | Fishery | -37.806 | -4.067 | - | 15 |
| 361 | Fishery | -37.806 | -4.109 | - | 15 |
| 362 | Fishery | -37.718 | -4.072 | - | 15 |
| 363 | Fishery | -36.125 | -4.992 | - | 15 |
| 364 | Fishery | -36.003 | -4.992 | - | 15 |
| 365 | Fishery | -35.957 | -4.936 | - | 15 |
| 366 | Fishery | -36.083 | -4.936 | - | 15 |
| 367 | Coral | -36.409 | -4.788 | - | 16 |
| 368 | Coral | -37 | -4.55 | - | 17 |
| 369 | Coral | -37.9 | -3.667 | - | 17 |
| 370 | Coral | -37.95 | -3.583 | - | 17 |
| 371 | Coral | -38.633 | -3.35 | - | 17 |
| 372 | Coral | -38.517 | -3.217 | - | 17 |
| 373 | Coral | -39.067 | -2.867 | - | 17 |
| 374 | Coral | -41.85 | -2.217 | - | 17 |
| 375 | Coral | -40.75 | -2.167 | - | 17 |
| 376 | Coral | -38.713 | -3.068 | - | 18 |
| 377 | Coral | -39.347 | -2.482 | - | 18 |
| 378 | Coral | -43.572 | -1.35 | - | 18 |
| 379 | Coral | -40.73 | -2.48 | - | 19 |
| 380 | Coral | -36.181 | -4.807 | - | 20 |
| 381 | Coral | -36.579 | -4.689 | - | 20 |
| 382 | Coral | -36.424 | -4.748 | - | 20 |
| 383 | Coral | -36.779 | -4.612 | - | 20 |
| 384 | Algae | -38.467 | -3.585 | - | 21 |
| 385 | Algae | -38.4 | -3.385 | - | 21 |
| 386 | Algae | -38.901 | -3 | - | 21 |
| 387 | Algae | -39.118 | -3.001 | - | 21 |
| 388 | Algae | -39.218 | -2.936 | - | 21 |
| 389 | Algae | -39.318 | -2.883 | - | 21 |
| 390 | Algae | -39.433 | -2.818 | - | 21 |
| 391 | Algae | -39.784 | -2.551 | - | 21 |
| 392 | Algae | -39.851 | -2.502 | - | 21 |
| 393 | Algae | -40.068 | -2.385 | - | 21 |
| 394 | Algae | -40.202 | -2.336 | - | 21 |
| 395 | Algae | -40.702 | -2.317 | - | 21 |
| 396 | Algae | -40.735 | -2.1 | - | 21 |
| 397 | Algae | -40.985 | -2.418 | - | 21 |
| 398 | Algae | -40.983 | -2.434 | - | 21 |
| 399 | Algae | -40.935 | -2.518 | - | 21 |
| 400 | Algae | -40.869 | -2.567 | - | 21 |
| 401 | Algae | -40.901 | -2.568 | - | 21 |
| 402 | Algae | -40.869 | -2.567 | - | 21 |
| 403 | Algae | -40.867 | -2.553 | - | 21 |
| 404 | Algae | -40.835 | -2.551 | - | 21 |
| 405 | Algae | -40.733 | -2.551 | - | 21 |
| 406 | Algae | -40.702 | -2.551 | - | 21 |
| 407 | Algae | -40.685 | -2.55 | - | 21 |
| 408 | Algae | -40.684 | -2.55 | - | 21 |
| 409 | Algae | -40.568 | -2.485 | - | 21 |
| 410 | Algae | -40.551 | -2.433 | - | 21 |
| 411 | Algae | -40.284 | -2.352 | - | 21 |
| 412 | Algae | -40.269 | -2.518 | - | 21 |
| 413 | Algae | -39.85 | -2.417 | - | 21 |
| 414 | Algae | -39.851 | -2.501 | - | 21 |
| 415 | Algae | -39.818 | -2.567 | - | 21 |
| 416 | Algae | -39.783 | -2.502 | - | 21 |
| 417 | Algae | -39.735 | -2.451 | - | 21 |
| 418 | Algae | -39.518 | -2.635 | - | 21 |
| 419 | Algae | -39.585 | -2.701 | - | 21 |
| 420 | Algae | -39.6 | -2.651 | - | 21 |
| 421 | Algae | -39.417 | -2.55 | - | 21 |
| 422 | Algae | -39.317 | -2.734 | - | 21 |
| 423 | Algae | -39.367 | -2.835 | - | 21 |
| 424 | Algae | -39.117 | -2.819 | - | 21 |
| 425 | Algae | -38.969 | -2.935 | - | 21 |
| 426 | Algae | -38.886 | -3.185 | - | 21 |
| 427 | Algae | -38.5 | -3.286 | - | 21 |
| 428 | Algae | -38.068 | -3.484 | - | 21 |
| 429 | Algae | -38.285 | -3.718 | - | 21 |
| 430 | Algae | -38.267 | -3.718 | - | 21 |
| 431 | Algae | -38.086 | -3.618 | - | 21 |
| 432 | Algae | -37.918 | -3.684 | - | 21 |
| 433 | Algae | -37.852 | -3.684 | - | 21 |
| 434 | Algae | -37.867 | -3.783 | - | 21 |
| 435 | Algae | -37.952 | -3.786 | - | 21 |
| 436 | Algae | -37.967 | -3.801 | - | 21 |
| 437 | Algae | -37.719 | -3.868 | - | 21 |
| 438 | Algae | -37.553 | -3.901 | - | 21 |
| 439 | Algae | -37.636 | -3.984 | - | 21 |
| 440 | Algae | -37.667 | -4.017 | - | 21 |
| 441 | Algae | -37.7 | -4.052 | - | 21 |
| 442 | Algae | -37.551 | -4.2 | - | 21 |
| 443 | Algae | -37.534 | -4.201 | - | 21 |
| 444 | Algae | -37.6 | -4.017 | - | 21 |
| 445 | Algae | -37.567 | -4.019 | - | 21 |
| 446 | Algae | -37.551 | -4.035 | - | 21 |
| 447 | Algae | -37.517 | -4.069 | - | 21 |
| 448 | Algae | -37.352 | -4.219 | - | 21 |
| 449 | Algae | -37.334 | -4.219 | - | 21 |
| 450 | Algae | -37.319 | -4.136 | - | 21 |
| 451 | Algae | -37.369 | -4.085 | - | 21 |
| 452 | Algae | -37.517 | -3.968 | - | 21 |
| 453 | Algae | -37.519 | -3.936 | - | 21 |
| 454 | Algae | -37.318 | -4.135 | - | 21 |
| 455 | Algae | -37.301 | -4.184 | - | 21 |
| 456 | Algae | -37.202 | -4.301 | - | 21 |
| 457 | Algae | -37.135 | -4.351 | - | 21 |
| 458 | Algae | -37.153 | -4.417 | - | 21 |
| 459 | Algae | -37.033 | -4.484 | - | 21 |
| 460 | Algae | -37.035 | -4.535 | - | 21 |
| 461 | Porifera | -36.118 | -4.857 | - | 22 |
| 462 | Porifera | -38.409 | -3.588 | - | 23 |
| 463 | Porifera | -41.334 | -2.333 | - | 24 |
| 464 | Porifera | -39.6 | -2.367 | - | 24 |
| 465 | Porifera | -39.067 | -2.867 | - | 24 |
| 466 | Porifera | -38.5 | -3.2 | - | 24 |
| 467 | Porifera | -37.067 | -4.45 | - | 24 |
| 468 | Porifera | -39.174 | -2.868 | - | 16 |
| 469 | Porifera | -38.959 | -3.031 | - | 16 |
| 472 | Porifera | -38.527 | -3.358 | - | 16 |
| 473 | Porifera | -38.409 | -3.588 | - | 16 |
| 474 | Porifera | -38.409 | -3.569 | - | 16 |
| 475 | Porifera | -38.392 | -3.598 | - | 16 |
| 476 | Porifera | -36.212 | -4.883 | - | 16 |
| 478 | Porifera | -39.17 | -2.868 | - | 25 |
| 479 | Porifera | -39.142 | -3.058 | - | 25 |
| 481 | Porifera | -38.954 | -3.031 | - | 25 |
| 482 | Porifera | -38.928 | -3.223 | - | 25 |
| 485 | Porifera | -38.527 | -3.359 | - | 25 |
| 486 | Porifera | -38.5 | -3.55 | - | 25 |
| 487 | Porifera | -40.783 | -2.333 | - | 26 |
| 488 | Porifera | -36.851 | -4.611 | - | 26 |
| 489 | Porifera | -36.837 | -4.626 | - | 26 |
| 490 | Porifera | -36.836 | -4.627 | - | 26 |
| 491 | Porifera | -36.822 | -4.624 | - | 26 |
| 492 | Porifera | -36.795 | -4.795 | - | 26 |
| 493 | Porifera | -36.767 | -4.626 | - | 26 |
| 494 | Porifera | -36.761 | -4.632 | - | 26 |
| 495 | Porifera | -36.756 | -4.799 | - | 26 |
| 496 | Porifera | -36.747 | -4.788 | - | 26 |
| 497 | Porifera | -36.746 | -4.788 | - | 26 |
| 498 | Porifera | -36.715 | -4.815 | - | 26 |
| 499 | Porifera | -36.707 | -4.808 | - | 26 |
| 500 | Porifera | -36.656 | -4.791 | - | 26 |
| 501 | Porifera | -36.655 | -4.791 | - | 26 |
| 502 | Porifera | -36.652 | -4.799 | - | 26 |
| 503 | Porifera | -36.652 | -4.749 | - | 26 |
| 504 | Porifera | -36.649 | -4.669 | - | 26 |
| 505 | Porifera | -36.646 | -4.749 | - | 26 |
| 506 | Porifera | -36.567 | -4.7 | - | 26 |
| 507 | Porifera | -36.466 | -4.976 | - | 26 |
| 508 | Porifera | -36.461 | -4.972 | - | 26 |
| 509 | Porifera | -36.451 | -4.848 | - | 26 |
| 510 | Porifera | -36.446 | -4.961 | - | 26 |
| 511 | Porifera | -36.444 | -4.802 | - | 26 |
| 512 | Porifera | -36.434 | -4.805 | - | 26 |
| 513 | Porifera | -36.42 | -5.001 | - | 26 |
| 514 | Porifera | -36.395 | -4.821 | - | 26 |
| 515 | Porifera | -36.392 | -4.79 | - | 26 |
| 516 | Porifera | -36.391 | -4.822 | - | 26 |
| 517 | Porifera | -36.389 | -4.791 | - | 26 |
| 518 | Porifera | -36.387 | -4.792 | - | 26 |
| 519 | Porifera | -36.321 | -4.964 | - | 26 |
| 520 | Porifera | -36.305 | -4.797 | - | 26 |
| 521 | Porifera | -36.295 | -4.79 | - | 26 |
| 522 | Porifera | -36.294 | -4.791 | - | 26 |
| 523 | Porifera | -36.294 | -4.789 | - | 26 |
| 524 | Porifera | -36.292 | -4.838 | - | 26 |
| 525 | Porifera | -36.25 | -4.961 | - | 26 |
| 526 | Porifera | -36.248 | -4.957 | - | 26 |
| 527 | Porifera | -36.246 | -4.954 | - | 26 |
| 529 | Porifera | -36.212 | -4.883 | - | 26 |
| 530 | Porifera | -36.21 | -4.969 | - | 26 |
| 531 | Porifera | -36.208 | -4.969 | - | 26 |
| 532 | Porifera | -36.203 | -4.833 | - | 26 |
| 533 | Porifera | -36.199 | -4.916 | - | 26 |
| 534 | Porifera | -36.199 | -4.809 | - | 26 |
| 535 | Porifera | -36.198 | -4.84 | - | 26 |
| 536 | Porifera | -36.183 | -4.886 | - | 26 |
| 537 | Porifera | -36.117 | -4.95 | - | 26 |
| 538 | Porifera | -38.959 | -3.031 | - | 27 |
| 540 | Porifera | -36.744 | -4.645 | - | 28 |
| 541 | Porifera | -37.634 | -3.909 | - | 28 |
| 542 | Porifera | -41.333 | -2.333 | - | 29 |
| 543 | Porifera | -40.75 | -2.167 | - | 29 |
| 544 | Porifera | -39.883 | -2.183 | - | 29 |
| 545 | Porifera | -39.733 | -2.267 | - | 29 |
| 546 | Porifera | -38.6 | -3.25 | - | 29 |
| 547 | Porifera | -38.517 | -3.217 | - | 29 |
| 548 | Porifera | -37 | -4.55 | - | 29 |
| 549 | Porifera | -37.233 | -4.383 | - | 29 |
| 550 | Porifera | -37.367 | -4.233 | - | 29 |
| 551 | Porifera | -37.55 | -4.017 | - | 29 |
| 552 | Porifera | -38.1 | -3.533 | - | 29 |
| 553 | Porifera | -38.967 | -2.983 | - | 29 |
| 554 | Porifera | -39.067 | -2.867 | - | 29 |
| 555 | Porifera | -39.167 | -2.683 | - | 29 |
| 556 | Porifera | -39.417 | -2.483 | - | 29 |
| 557 | Porifera | -39.6 | -2.367 | - | 29 |
| 558 | Porifera | -38.233 | -3.3 | - | 29 |
| 559 | Porifera | -35.6 | -4.717 | - | 29 |

**References**

1 Almeida, L. G. Caracterização das áreas de pesca artesanal de lagosta na Praia da Redonda, Icapuí-Ce, thesis, Universidade Federal do Ceará (2010).

2 Cabral Neto, I. Beachrocks do Rio Grande do Norte: correlação entre os depósitos costeiros e os de zona costa-afora com base na faciologia, petrografia e diagênese, thesis, Universidade Federal do Rio Grande do Norte (2011).

3 Freitas, J. E. P. & Lotufo, T. M. C. Reef fish assemblage and zoogeographic affinities of a scarcely known region of the western equatorial Atlantic. J. Mar. Biol. Assoc. United Kingdom. 95, 623–633 (2015).

4 Freitas, J. E. P., Araujo, M. E. & Lotufo, T. M. C. Composition and structure of the ichthyofauna in a marine protected area in the western equatorial Atlantic: A baseline to support conservation management. Reg. Stud. Mar. Sci. 25, 100488 (2019).

5 Garcia Júnior, J. Inventário das espécies de peixes da costa do estado do Rio Grande do Norte e aspectos zoogeográficos da ictiofauna recifal do oceano atlântico, thesis, Universidade Federal do Rio Grande do Norte (2006).

6 Martins, F. A. S. Ictiofauna associada a recifes naturais e artificais em uma reserva extrativista marinha, thesis, Universidade Federal do Ceará (2019).

7 Monteiro, L. H. U. Feições superficiais da plataforma continental cearense entre o litoral de Fortaleza e Icapuí, thesis, Universidade Federal de Pernambuco (2011).

8 Morais, J. O., Ximenes Neto, A. R., Pessoa, P. R. S. & Souza, L. P. Morphological and sedimentary patterns of a semi-arid shelf, Northeast Brazil. Geo-Marine Lett., 1–8 (2019).

9 Pantalena, A. F. Mergulho recreativo na região metropolitana de Fortaleza (NE, Brasil): subsídios para o desenvolvimento sustentável, thesis, Universidade Federal do Ceará (2017).

10 Silva, L. L. N., Gomes, M. P. & Vital, H. The Açu Reef morphology, distribution, and inter reef sedimentation on the outer shelf of the NE Brazil equatorial margin. Cont. Shelf Res. 160, 10–22 (2018).

11 Soares, M. O., Davis, M., Paiva, C. C. & Carneiro, P. B. M. Mesophotic ecosystems: coral and fish assemblages in a tropical marginal reef (northeastern Brazil). Mar. Biodivers., 1–6 (2016).

12 Sousa Filho, L. M. As aventuras de um pescador sub (ABC Editora, Fortaleza, 2002).

13 Braga, M. S. C. Embarcações a vela do litoral do Estado do Ceará construção, construtores, navegação e aspectos pesqueiros, thesis, Universidade Federal do Ceará (2014).

14 Nóbrega, M., Kinas, P. G., Ferrandis, E. & Lessa, R. P. T. Distribuição espacial e temporal da guaiúba Ocyurus chrysurus (Bloch, 1791)(Teleostei, Lutjanidae) capturada pela frota pesqueira artesanal na região nordeste do Brasil. Panam. J. Aquat. Sci. 4, 17–34 (2009).

15 Nóbrega, M. F. & Lessa, R. P. Descrição e composição das capturas da frota pesqueira artesanal da região nordeste do Brasil. Arq. Ciências do Mar. 40, 64–74 (2007).

16 Scientific collection - MNRJ

17 Scientific collection - MOUFPE

18 Scientific collection - UFC

19 Scientific collection - USNM

20 Cordeiro, R. T., Neves, B. M., Kitahara, M. V., Arantes, R. C. & Perez, C. D. First assessment on Southwestern Atlantic equatorial deep-sea coral communities. Deep. Res. Part I Oceanogr. Res. Pap. 163, 103344 (2020).

21 Liberato, M. A. F. Algas marinhas de profundidade, prospectadas durante a operação Geomar XVIII, thesis, Universidade Federal do Ceará (1982).

22 Barros, L. V., Santos, G. G. & Pinheiro, U. Clathria (Clathria) Schmidt, 1862 from Brazil with description of a new species and a review of records (Poecilosclerida: Demospongiae: Porifera). Zootaxa. 3640, 284–295 (2013).

23 Bezerra, L. E. A. & Coelho, P. A. Crustáceos decápodos associados a esponjas no litoral do Estado do Ceará, Brasil. Rev. Bras. Zool. 23, 699–702 (2006).

24 Borojevic, R. & Peixinho, S. Éponges calcaires du Nord-Nord-Est du Brésil. Bull. du Muséum Natl. d’Histoire Naturelle. 279:, 987–1036 (1976).

25 Lotufo, T. M. C., Hajdu, E. M., Santos, C. P., Ribeiro, C. A., Joca, I. R., Silva, F. F., Rocha, M. F. & Cavalcanti, G. H. Sessile epifauna of Ceara's shelf - high dominance of sponges in Abstracts of the 7th International Sponge Symposium (Museu Nacional do Rio de Janeiro, Rio de Janiro, 2006), pp. 123.

26 Muricy, G. Biodiversidade marinha de Bacia Potiguar: Porifera (Museu Nacional, Rio de Janeiro, 2008).

27 Pinheiro, U. S. "Contribuições à taxonomia e biogeografia das Esponjas de águas continentais Brasileiras", thesis, Universidade Federal do rio de Janeiro, 220f (2007).

28 Santos, J. P., Mothes, B., Tenório, D. O. & Cantarelli, J. Porifera (DEMOSPONGIAE, CALCAREA) entre os estados do Ceará e Pernambuco, Brasil. Taxon. e Distrib. Trab. do Inst. Oceanogr. da Univ. Fed. Pernambuco. 27, 49–60 (1999).

29 Nascimento, E. F. "Taxonomia das esponjas marinhas do Nordeste brasileiro: Expedição Canopus (1965-1966)", thesis, Universidade Federal de Pernambuco (2019).

**Supplementary Table S2. List of fish species captured in 35 bottom longline fisheries along the outer continental shelf and shelf slope of the Brazilian Semi-Arid Coast**. N = non-reef species, RES = reef resident, PE-OCA = pelagic occasionally on reefs, BE-OCA = demersal occasionally on reefs, NA = data not available (i.e., species was not weighted and/or identified onboard).

| **TAXON** | | **TYPE*** | **% BIOMASS** | | **FREQ** | |
| --- | --- | --- | --- | --- | --- | --- |
| **ACTINOPTERYGII** | | | | | | |
| **CARANGIDAE** | |  |  | |  | |
| *Caranx hippos* | | RES | NA | | NA | |
| *Caranx lugubris* | | RES | 0.89 | | 21 | |
| *Seriola* spp. | | RES | 0.76 | | 14 | |
| **CORYPHAENIDAE** | |  |  | |  | |
| *Coryphaena hippurus* | | PE-OCA | 0.05 | | 4 | |
| **GEMPYLIDAE** | |  |  | |  | |
| *Ruvettus prettiosus* | | N | 0.14 | | 5 | |
| **ISTIOPHORIDAE** | |  |  | |  | |
| *Istiophorus* spp. | | PE-OCA | 0.05 | | 1 | |
| **LUTJANIDAE** | |  |  | |  | |
| *Lutjanus analis / L. jocu / L. purpureus / L. synagris* | | RES | 9.76 | | 35 | |
| **MALACANTHIDEA** | |  |  | |  | |
| *Lopholatilus villari* | | BE-OCA | 0.09 | | 1 | |
| **MEGALOPIDEA** | |  |  | |  | |
| *Megalops atlanticus* | | PE-OCA | 0.45 | | 6 | |
| **MURAENIDAE** | |  |  | |  | |
| *Gymnothorax* spp. | | RES | 0.31 | | 22 | |
| **RANCYCENTRIDEA** | |  |  | |  | |
| *Rachycentron canadum* | | PE-OCA | 1.18 | | 27 | |
| **SCOMBRIDEA** | |  |  | |  | |
| *Scomberomorus* spp. | | PE-OCA | 0.03 | | 3 | |
| *Thunnus* spp. | | PE-OCA | 0.02 | | 1 | |
| **SERRANIDAE** | |  |  | |  | |
| *Epinephelus itajara* | | RES | 0.79 | | 8 | |
| *Epinephelus morio / E. marginatus* | | RES | 1.84 | | 25 | |
| *Hyporthodus niveatus* | | RES | 0.14 | | 3 | |
| *Mycteroperca bonaci* | | RES | 10.66 | | 35 | |
| *Mycteroperca* sp. | | RES | NA | | NA | |
| **XIPHIIDAE** | |  |  | |  | |
| *Xiphias gladius* | | PE-OCA | NA | | NA | |
| **ELASMOBRANCHII** | | | | | | |
| **CARCHARHINIDAE** |  | | |  | |  |
| *C. acronotus / Rizoprionodon lalandii / R. porosus* | RES / BE-OCA | | | 2.99 | | 29 |
| *Galeocerdo cuvier* | BE-OCA | | | 2.09 | | 18 |
| *Carcharhinus plumbeus / C. longimanus*  *C. signatus / C. obscurus / C. falciformis*  *C. leucas / C. limbatus* | PE-OCA /  BE-OCA | | | 21.13 | | 34 |
| *Negaprion brevirostris* | RES | | | 0.09 | | 2 |
| **DASYATIDEA** |  | | |  | |  |
| *Hypanus berthalutzae/ H. guttatus* | RES / BE-OCA | | | 20.64 | | 32 |
| *Hypanus marianae* | BE-OCA | | | 0.17 | | 1 |
| **GINGLIMOSTOMATIDAE** |  | | |  | |  |
| *Ginglymostoma cirratum* | RES | | | 18.54 | | 35 |
| **HEXANCHIDAE** |  | | |  | |  |
| Hexanchidae | BE-OCA | | | 0.07 | | 4 |
| **MOBULIDAE** |  | | |  | |  |
| *Mobula* spp. | PE-OCA | | | 0.13 | | 3 |
| **MYLIOBATIDEA** |  | | |  | |  |
| *Aetobatus narinari* | PE-OCA | | | 0.21 | | 10 |
| **SPHYRNIDAE** |  | | |  | |  |
| *Sphyrna lewini / S. mokarran / S. zygaena* | PE-OCA | | | 6.73 | | 25 |
| **SQUALIDAE** |  | | |  | |  |
| *Squalus* spp. | PE-OCA | | | 0.07 | | 5 |
| **RHINOPTERIDAE** |  | | |  | |  |
| *Rhinoptera* spp. | PE-OCA | | | NA | | NA |

*According to Pinheiro, H. T. et al. South-western Atlantic reef fishes: Zoogeographical patterns and ecological drivers reveal a secondary biodiversity centre in the Atlantic Ocean. Divers. Distrib. 24, 951–965 (2018).
