# Supplementary material for: Interconnected marine habitats form a single continental-scale reef system in South America
Source: Sci Rep. 2022 Oct 17;12:17359. doi: 10.1038/s41598-022-21341-x (PMC9576765; doi:10.1038/s41598-022-21341-x)
Supplement: Supplementary file 3 — Supplementary Information 3. [file 41598_2022_21341_MOESM3_ESM.docx]

**Supplementary Table S2. List of fish species captured in 35 bottom longline fisheries along the outer continental shelf and shelf slope of the Brazilian Semi-Arid Coast**. N = non-reef species, RES = reef resident, PE-OCA = pelagic occasionally on reefs, BE-OCA = demersal occasionally on reefs, NA = data not available (i.e., species was not weighted and/or identified onboard).

**Supplementary Table S1.**  List of georeferenced localities with either known rocky substrates (i.e. Reef), reef-fish fishing grounds (i.e. fisheries) or epilithic benthic assemblages (i.e. Porifera, Coral and Algae) along the Brazilian semi-arid coast.

**Fig. S1. Ordination analyses (Sammon mapping) of South Atlantic reef systems and biogeographic regions based on the Sørensen dissimilarities among their marine biotas.** Analysis on a dataset with 2412 reef species, published by 47. A minimum spanning tree was superimposed to the ordination graph in order to highlight putative connection pathways among regions 48,49. Note that SAC is between ARS and ERS in the majority of cases. ARS = Amazon Reef System, SAC = Brazilian Semi-Arid Coast reef system, ERS = Eastern Brazilian reef system.

**Fig. S2. Ordination analyses (Sammon mapping) of South Atlantic reef systems and biogeographic regions based on the Sørensen dissimilarities among their marine biotas.** Analysis on a dataset with 8375 marine species, published by 46. A minimum spanning tree was superimposed to the ordination graph in order to highlight putative connection pathways among regions 48,49. Note that SAC is between ARS and ERS in half of the cases. ARS = Amazon Reef System, SAC = Brazilian Semi-Arid Coast reef system, ERS = Eastern Brazilian reef system.

**Video S1. Remotely Operated Underwater Vehicle (ROV) video footage of benthic assemblages along the outer continental shelf (~60m deep) of the Brazilian semi-arid coast**. Note the likely rocky nature of the substrate.
